# RTEL1 influences the abundance and localization of TERRA RNA

**DOI:** 10.1038/s41467-021-23299-2

**Published:** 2021-05-21

**Authors:** Fiorella Ghisays, Aitor Garzia, Hexiao Wang, Claudia Canasto-Chibuque, Marcel Hohl, Sharon A. Savage, Thomas Tuschl, John H. J. Petrini

**Affiliations:** 1 Molecular Biology Program, Memorial Sloan-Kettering Cancer Center, New York, NY USA; 2 Laboratory for RNA Molecular Biology, The Rockefeller University, New York, NY USA; 3 Clinical Genetics Branch, Division of Cancer Epidemiology and Genetics, National Cancer Institute, Rockville, MD USA

**Keywords:** RNA, Chromosomes

## Abstract

Telomere repeat containing RNAs (TERRAs) are a family of long non-coding RNAs transcribed from the subtelomeric regions of eukaryotic chromosomes. TERRA transcripts can form R-loops at chromosome ends; however the importance of these structures or the regulation of TERRA expression and retention in telomeric R-loops remain unclear. Here, we show that the RTEL1 (Regulator of Telomere Length 1) helicase influences the abundance and localization of TERRA in human cells. Depletion of RTEL1 leads to increased levels of TERRA RNA while reducing TERRA-containing R loops at telomeres. In vitro, RTEL1 shows a strong preference for binding G-quadruplex structures which form in TERRA. This binding is mediated by the C-terminal region of RTEL1, and is independent of the RTEL1 helicase domain. RTEL1 binding to TERRA appears to be essential for cell viability, underscoring the importance of this function. Degradation of TERRA-containing R-loops by overexpression of RNAse H1 partially recapitulates the increased TERRA levels and telomeric instability associated with RTEL1 deficiency. Collectively, these data suggest that regulation of TERRA is a key function of the RTEL1 helicase, and that loss of that function may contribute to the disease phenotypes of patients with *RTEL1* mutations.

## Abstract (web summary)

Long non coding RNA TERRA transcripts can form R-loops at chromosome ends. Here, the authors reveal a role for the helicase RTEL in affecting TERRA levels and localization.

## Introduction

RTEL1 is an (Fe-S) cluster containing helicase that belongs to the DEAH superfamily 2 (SF2) and specifies 5’-3’ helicase activity^1,2^. The *RTEL1* gene was first identified in a four-gene cluster on chromosome 20 found to be amplified in gastrointestinal cancers (then called NHL)^3^. The gene was subsequently identified as a regulator of telomere length in the mouse and renamed RTEL1 (for Regulator of Telomere Length)^4^.

RTEL1 plays diverse functional roles that bear upon several aspects of genome integrity and gene expression. RTEL1 influences telomere stability by facilitating replication at telomeres, possibly by removing secondary structures that would otherwise inhibit replication fork progression^5,6^. In addition, it influences genome stability via its roles in regulating DNA replication and DNA recombination in mammals, *C. elegans*, and in plants^7,8^. More recently, RTEL1 has been shown to promote MiDAS, a mitotic DNA synthesis process that occurs in common fragile sites and other late replicating genomic loci^9,10^. Finally, the protein also appears to influence RNA trafficking, as cells from patients with inherited *RTEL1* mutations exhibit aberrant localization of pre-U2 RNA and associated ribonucleoproteins (RNPs)^11^.

Germline mutations in *RTEL1* underlie Dyskeratosis Congenita (DC) and the more severe variant, Hoyeraal-Hreidarsson syndrome (HH)^12,13^. At the cellular level, these diseases are associated with very short and heterogeneous telomeres, genomic instability, and sensitivity to clastogens, consistent with the role of RTEL1 in preserving genome integrity^14,15^. Some disease-causing *RTEL1* alleles exhibit autosomal dominant behavior, suggesting that the protein may function in a dimeric or multimeric assembly^16–19^.

Telomeric repeat containing RNAs (TERRAs) are RNA polymerase II transcripts emanating from subtelomeric regions that extend into the TTAGGG telomeric repeats^20–23^. TERRA transcripts are heterogeneous in size and can be associated with chromosome ends by forming R-loops either co-transcriptionally or post-transcriptionally. In the latter circumstance, the TERRA RNA may be recruited via binding to telomeric proteins^20,22,24–29^.

TERRA is transcribed from at least a subset of chromosome ends, and the mechanisms of regulation may differ between chromosomes. TERRA binds to telomeres as well as interstitial sites where it can influence gene expression^22,24,30,31^. Despite this broad distribution of binding sites, the most prominent role of TERRA is at the telomere. Depletion of TERRA leads to myriad indices of telomere dysfunction such as the loss of telomeric DNA, the formation of telomere damage-associated foci (TIFs)^32^, and telomere driven chromosome aberrations. Notably, TERRA interacts with, and appears to antagonize the functions of the chromatin remodeler ATRX, which plays a key role in modulating telomeric chromatin structure^24,33,34^.

Owing to its G-rich sequence, TERRA can form stable, four-stranded structures known as G-quadruplexes in vitro^35–37^. G-quadruplex structures can form in both G-rich DNA and RNA sequences in vitro under physiological conditions, and would constitute barriers to protein translocation during DNA replication, transcription, and mRNA translation if present in cells^38^. There are dedicated protein machineries with the capacity to unfold G-quadruplex structures in mammalian cells^39^. However, it is not clear whether TERRA forms G-quadruplex structures in vivo and if so, whether those structures are biologically relevant.

Here, we present evidence that RTEL1 influences the levels and localization of TERRA via direct physical interaction. We identified an RNA-binding domain in the RTEL1 C-terminus that exhibits a strong preference for G-quadruplex folded TERRA RNA over unfolded RNA. RTEL1 deficiency causes a dramatic increase in TERRA levels, but reduced localization of TERRA RNA to chromosome ends. Our data thus reveal a previously unrecognized function of RTEL1 in regulating the disposition of the TERRA RNA. The data are consistent with the observation that misregulation of TERRA compromises telomere maintenance, and suggest an additional mechanism by which *RTEL1* hypomorphism may cause telomere instability and contribute to the clinical phenotypes of DC and HH^24,25,30,40–46^.

## Results

Homozygosity of the *RTEL1*^*R1264H*^ pathogenic variant causes HH, which occurs as a heterozygous founder mutation in the Ashkenazi Jewish population^15^. The mutation falls within the RING domain at the protein’s C-terminus, and its implication as an underlying cause of HH provided the first evidence of the RING domain’s functional significance^15^. Typically, the RING domain structure is dependent upon the coordination of Zn^2+^ atoms via cysteine and histidine residues. The R→H change in the *RTEL1*^*R1264H*^ RING domain introduces an additional potential Zn^2+^ coordination partner that may alter the RING domain’s structure.

### Domain analysis of RTEL1

**Fig. 1:**
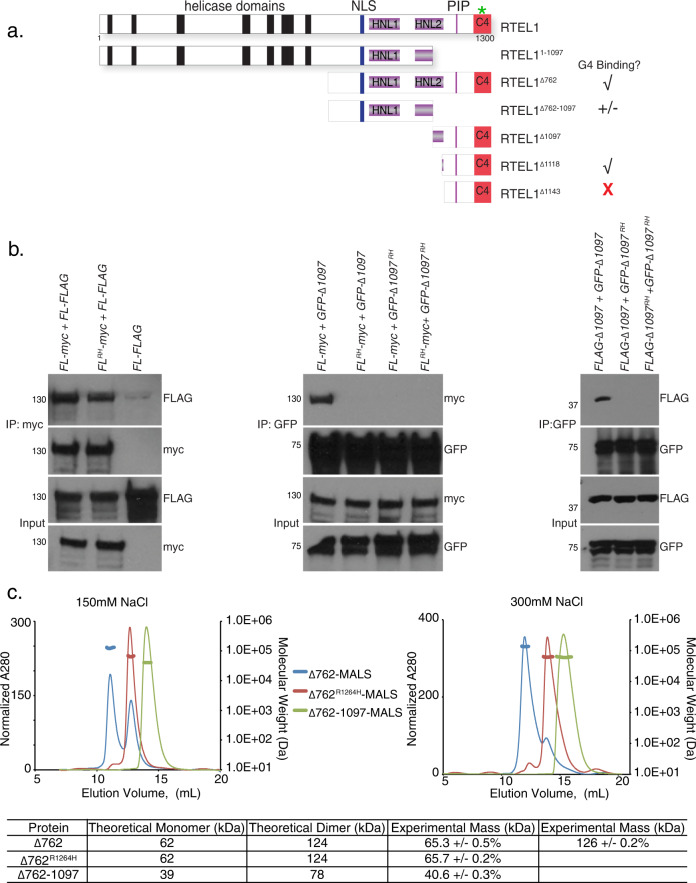
Domain Analysis of RTEL1. **a** Schematic representation of RTEL1 proteins and constructs used in this study. The location of the *RTEL1*^*R1264H*^ mutation is indicated with a green star and G4 binding is illustrated. **b** RTEL1 interacts with itself in cells and the *RTEL1*^*R1264H*^ mutation disrupts interactions within the RING domain. Left, Myc immunoprecipitations from HEK293T cells co-expressing FLAG and myc-tagged *RTEL1 (FL)* and *RTEL1*^*R1264H*^ (*FL* ^*RH*^*)* were carried out. Immunoblotting with FLAG indicates co-IP of both *RTEL1* and *RTEL1*^*R1264H*^. Center, immunoblotting with myc indicates co-IP of RTEL1^∆1097^ (∆1097), but not RTEL1^∆1097 R1264H^ (∆1097 ^RH^) with GFP-tagged *RTEL1* and *RTEL1*^*R1264H*^. Right, immunoblotting with FLAG shows co-IP of RTEL1^∆1097^, but not RTEL1^∆1097R1264H^. Molecular weight markers, KDa are shown. All immunoblotting experiments were repeated at least three times with similar results. **c** Oligomeric states of RTEL1 proteins were determined by SEC-MALS analysis of RTEL1^∆762^ (blue), RTEL1^∆762R1264H^ (red), and RTEL1^∆762-1097^ (green) at 150 and 300 mM NaCl. Normalized A280 are shown and the calculated molecular mass is shown as a line in the corresponding color across each peak with the secondary scale on the right. The expected and calculated molecular weights are shown (see also Supplementary Fig. 1).

To examine the functional consequences of the *RTEL1*^*R1264H*^ mutation at the molecular level, we designed a series of constructs for the production of various RTEL1 protein domains (Fig. 1a). First, differentially tagged (myc and FLAG) wild type and *RTEL1*^*R1264H*^ full-length complementary DNAs (cDNAs) were co-expressed in HEK293 cells. FLAG-tagged RTEL1 was recovered in myc immunoprecipitates of both *RTEL1*^*R1264H*^ and wild type RTEL1, suggesting that the protein can form a higher order assembly (Fig. 1b, left).

This assembly is mediated by spatially distinct regions of RTEL1, one in the N-terminal helicase domain and the other in the C-terminal RING domain. The protein product of *FLAG*-*RTEL1*^*∆1097*^, which comprises the C-terminal 203 amino acids of RTEL1 including the PIP box and RING domain (Fig. 1a), co-immunoprecipitated with both full-length RTEL1 and itself. These interactions were abolished by the *RTEL1*^*R1264H*^ mutation (Fig. 1b, center and right), indicating that the RING domain constitutes an independent homotypic interaction domain that is rendered non-functional by the *RTEL1*^*R1264H*^ mutation. Full-length RTEL1^R1264H^ retains the ability to interact with wild type full-length RTEL1, indicating that the N-terminal half of RTEL1 also promotes oligomerization (Fig. 1b, left).

To characterize the C-terminal oligomerization domain of RTEL1, we began with RTEL1^∆762^ (which lacks the helicase domain but retains the harmonin N- like, PIP box, and RING domains of RTEL1), and designed variants, including the *RTEL1*^∆762*R1264H*^ mutation and various deletions within RTEL1^∆762^ that removed the RING domain and additional proximal sequences (Fig. 1a). We purified recombinant His-tagged RTEL1^∆762^ from *E.coli*. Size exclusion chromatography (SEC) revealed that RTEL1^∆762^ was present in two monodispersed peaks (Fig. 1c). Coupling the SEC with multiple angle light scattering (SEC-MALS) showed the molecular masses of the RTEL1^∆762^ peaks to be 65.3 ± 0.5% and 126 ± 0.2% kDa, respectively, corresponding to the expected monomer and dimer masses of RTEL1^∆762^ (62 kDa and 124 kDa) (Fig. 1c). The dimer peak was virtually absent in the RTEL1^∆762R1264H^ protein and the calculated molecular mass was 65.7 ± 0.2% kDa, corresponding to that of a monomer (Fig. 1c). The RTEL1^∆762-1097^ protein, which lacks the RING domain, is also a monomer (Fig. 1c).

SEC-MALS experiments showed that the RTEL1^∆762^ dimeric species was favored at higher ionic strength. Elevation of the salt concentration from 150 mM to 300 mM NaCl favored the RTEL1^∆762^ dimeric species but had no effect on the oligomeric state of either RTEL1^∆762R1264H^ or RTEL1^∆762-1097^ proteins (Fig. 1c). These results suggest that dimerization of the C-terminal RTEL1 domain is dependent on the RING domain in combination with hydrophobic interactions that are stabilized at higher ionic strength. Hence, the RING domain of RTEL1 is a dimerization domain and the *RTEL1*^*R1264H*^ mutation disrupts that function in vitro and in cells.

### Heterotypic interactions at the RTEL1 C-terminus

Having identified the homotypic interaction in the C-terminus of RTEL1, we reasoned that heterotypic protein interactions at the RTEL1 C-terminus could also provide insight regarding helicase independent functions of RTEL1, and shed light on the function(s) of the RING domain, as well as the consequences of the *RTEL1*^*R1264H*^ mutation. We used a BioID proximity dependent labeling system^47^ to identify RTEL1 C-terminal interacting proteins. This system uses the promiscuous biotin ligase BirA^R118G^ fused to the protein of interest. Upon addition of biotin to cells expressing BirA fusion proteins, biotin is covalently linked via lysine residues within a 10 nm radius of the BirA^R118G^ tag^48^, and biotinylated proteins can subsequently be purified via streptavidin binding. The biotin-labeled proteins can then be identified by mass spectrometry^47^.

BirA^R118G^ was fused to the C-terminus of full-length RTEL1 and RTEL1^1-1097^, from which part of the second Harmonin N-like repeat, the PIP box, and the RING domain are deleted (see Fig. 1a and Supplementary Fig. 1A). RTEL1 is normally located in the nucleus and fusion to BirA did not change its intracellular localization (Supplementary Fig. 1B). Nevertheless, the biotin-labeled proteins identified in this experiment included numerous RNA-binding proteins that function in the cytoplasm. These RNA-binding proteins were enriched by interaction with full-length RTEL1 relative to RTEL11–1097. Eighteen proteins obtained in our screen were also recovered in a screen to identify proteins bound to the telomeric RNA, TERRA^28^ (Supplementary Fig. 1C). Additionally, SFPQ and NONO have been recently characterized as TERRA-binding proteins and were among the top hits in our screen^49^. RTEL1 was also found in a separate screen for TERRA interacting proteins^24^.

### The RTEL1 C-terminal domain preferentially binds TERRA RNA

**Fig. 2:**
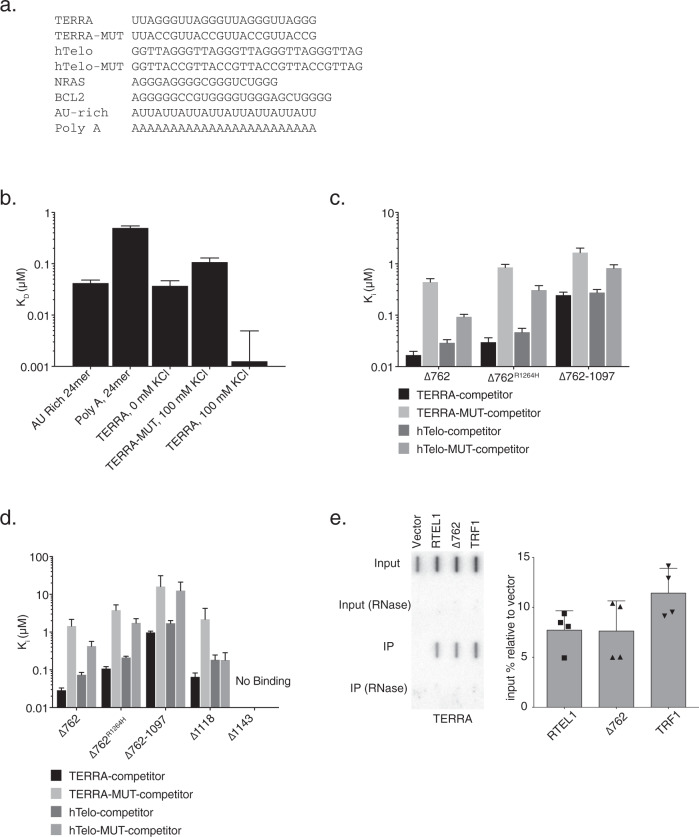
The RTEL1 C-terminal Domain Binds TERRA and other G-quadruplex Structures. **a** Sequences of DNA and RNA substrates used in this study. **b** Nucleic acid binding of RTEL1 proteins lacking the helicase domains was monitored by fluorescence anisotropy. Bar graph shows dissociation constants (*K*_D_) for TERRA and TERRA-MUT RNAs folded in the presence and absence of KCl and, AU-rich, and polyA RNA controls. **c** Increasing concentrations of the indicated oligonucleotides were added to reactions containing RTEL1 proteins and a 24mer FAM-TERRA-MUT RNA at 200 nM. Bar graphs depict apparent dissociation constants (*K*_i_) derived by competition of the bound FAM-TERRA-MUT by the indicated oligonucleotides using fluorescence anisotropy. **d** *K*_i_’s of the indicated RTEL1 deletion constructs were derived by competition studies of FAM-TERRA-MUT and indicated RTEL1 proteins at 1 µM as in panel C. No binding signal was observed for the RTEL1^∆1143^ protein. All binding assays were conducted in triplicate, mean and standard deviation are shown. **e** RNA IP assays were done in HEK293T cells transfected with FLAG-tagged RTEL1, ∆762, TRF1 or vector only control. Precipitated nucleic acids were treated with DNAse I for 1 h at 37 °C. IP RNA was analyzed by slot blotting and detected by autoradiography using a telomeric probe. Quantitation of four independent experiments with the mean and standard deviation of relative RNA-IP normalized to the vector only control is shown. Five percent of the input is shown in the top two rows. RNAse treatment is indicated (see also Supplementary Figs. 2–7).

We did not observe direct interactions between RTEL1 and the TERRA-binding proteins previously identified. Instead, we found that RTEL1 bound TERRA directly in vitro via a domain in the C-terminus. We assayed binding of RTEL1^∆762^ (Fig. 1a) to TERRA by fluorescence anisotropy. First, we determined the dissociation constant (*K*_D_) for fluorescein labeled G-quadruplex containing TERRA RNA (TERRA), a mutant TERRA RNA that is unable to form the G-quadruplex structure (TERRA-MUT), and TERRA RNA not folded into a G-quadruplex (Fig. 2a)^36^. TERRA RNA and the analogous DNA molecules were heated to 95 °C then slowly cooling them in 50 mM KCl. The presence of G-quadraplex structures in these substrates was validated by gel shift and circular dichroism as previously described^36,50^. Two RNAs unrelated to TERRA that were either unstructured (AU-rich) or differently structured (PolyA) were also examined^51^ (Fig. 2b). Fifty nanomolar RNA was incubated with increasing concentrations of RTEL1^∆762^ (0–5 µM) and incubated for 30 min at room temperature. RTEL1^∆762^ bound to all tested RNAs and showed a striking preference for the folded TERRA RNA, exhibiting a 500-fold preference over the PolyA RNA, and 100-fold over the TERRA-MUT substrate incapable of forming G-quadruplexes (Fig. 2b and Supplementary Fig. 2A).

The *K*_D_ for RTEL1^∆762^ binding to TERRA (<10 nM) is at the detection limit for anisotropy, which may compromise the accuracy of the measurement. As an alternative to direct determination of *K*_D_, we again used fluorescence anisotropy to measure the ability of substrates to compete with TERRA-MUT RNA and thereby derive *K*_i_ values as a surrogate for the apparent *K*_D_. RTEL1^∆762^, RTEL1^∆762R1264H^, and RTEL1^∆762-1097^ proteins (Fig. 1a) were pre-incubated with TERRA-MUT RNA (Fig. 2a) for 30 min to allow protein–RNA complexes to form, followed by addition of increasing amounts of wild type TERRA RNA or DNA competitor substrates. The *K*_i_ of RTEL1^∆762^ for the folded TERRA RNA was 17 ± 3 nM, 26-fold greater affinity than binding to TERRA-MUT RNA (*K*_i_ 442 ± 0.74) (Fig. 2c and Supplementary Fig. 2B).

RTEL1 bound G strand telomeric DNA (hTelo) with similar affinity to TERRA RNA; however, the preference for folded telomeric DNA relative to unfolded DNA (hTelo-MUT) is diminished by approximately threefold. The *K*_i_ for RTEL1^∆762^ binding hTelo DNA was 29 ± 4 nM, and for hTelo-MUT DNA (*K*_i_ 93 ± 11 nM) (Fig. 2c and Supplementary Fig. 2B). Hence, this domain of RTEL1 discriminates G4 from other RNA structures to greater extent than the same structures in DNA. Within the context of telomeric chromatin, it is unclear whether G4 DNA and RNA structures co-exist, but the selectivity of RTEL1 for folded RNA may be relevant to its telomeric functions.

### The G4-binding domain of RTEL1

To identify the location of the G4-binding domain in the RTEL1 C-terminus, we used fluorescence anisotropy as above with fragments of RTEL1^∆762^ (Fig. 1a). The RTEL1^∆762R1264H^ protein displayed similar binding affinities to the wild type RTEL1^∆762^ protein with only a two- to fourfold difference in *K*_i_ values (Fig. 2c and Supplementary Fig. 3). These data indicate that RNA/DNA binding is not directly mediated by the RING domain. The decreased apparent affinity of RTEL1^∆762R1264H^ likely reflects the difference in valency of the TERRA-binding domain in monomeric vs. dimeric RTEL1^∆762^.

Deletion of the C-terminal 230 residues of ∆762 (resulting in RTEL1^∆762-1097^ protein) reduced binding affinities by approximately 15-fold (Fig. 2c and Supplementary Fig. 2B) and led to a reduced preference for TERRA—6.7-fold relative to the 26.4-fold preference for TERRA in the wild type protein. Unlike binding to RTEL1^∆762^, the residual binding activity of RTEL1^∆762-1097^ was acutely sensitive to salt concentration (Supplementary Fig. 4). The binding affinity and selectivity for G4 binding of the RTEL1^∆1118^ protein product was similar to RTEL1^∆762^ (Fig. 2d), whereas binding was largely absent for RTEL1^∆1143^ (Fig. 2d and Supplementary Figs. 3 and  5). These data suggest that G4 binding is primarily specified by residues between amino acid 1097 and the RING domain (Fig. 1a).

As RTEL1 functions affect interstitial as well as telomeric chromosomal sites, we asked whether RTEL1 bound to G4 structures forming in RNA encoded from interstitial loci. We used two RNAs predicted to form G4 structures that fall within the 5’ untranslated regions of NRAS and BCL2 (Fig. 2a)^36^. RTEL1^∆762^, RTEL1^∆762R1264H,^ and RTEL1^∆762-1097^ proteins bound both NRAS and BCL2 RNAs with similar affinities to TERRA (Supplementary Fig. 6a, b).

### RTEL1 binds RNA in cells

To determine whether RTEL1 bound RNA in cells, we performed 4-thiouridine (4SU) photoactivatable ribonucleoside-enhanced crosslinking and immunoprecipitation (PAR-CLIP)^52^. FLAG immunoprecipitation from HEK293 cells overexpressing FLAG-HA-tagged RTEL1 and RTEL1^∆762^ was performed followed by RNAse treatment. RNA within immunoprecipitated complexes was 5’ end-radiolabeled with polynucleotide kinase and visualized by autoradiography. Both full-length RTEL1 and RTEL1^∆762^ immunoprecipitates contained radiolabeled RNA (Supplementary Fig. 6d, e). We were unable to obtain reliable RNA sequencing data from the material in the RTEL1 PAR-CLIP, possibly due to the structure of the RNA(s) bound in that context.

To determine if the cellular RNAs bound by RTEL1 included TERRA, we performed RNA-immunoprecipitations (RNA-IP) of overexpressed FLAG-RTEL1, FLAG-RTEL1^∆762^, and FLAG-TRF1 (as a positive control for a TERRA-binding protein^29,53^). The precipitated nucleic acids were treated in vitro with DNAseI, and RNAseA (where indicated). The signal was abolished upon pre treatment with RNAseA but resistant to DNAseI treatment confirming the specificity for RNA in this assay. The pretreated nucleic acids were slot blotted, probed for telomere repeats and other RNAs then visualized by autoradiography (Fig. 2e and Supplementary Fig. 7B). Expression levels of the immunoprecipitated proteins are shown in (Supplementary Fig. 7A). Consistent with our biochemical studies we found that RTEL1 and RTEL1^∆762^ bind TERRA, indicating that at least in part, the RNAs identified in PAR-CLIP experiments include TERRA (Fig. 2; Supplementary Figs. 6D, E and  7A, B). Also consistent with our in vitro studies, RTEL1 immunoprecipitations recovered *MYC* RNA (which has a G- quadruplex forming sequence its promoter region^54^) and other RNAs (Supplementary Figs. 6D, E and  7B).

### RTEL1 influences TERRA levels

**Fig. 3:**
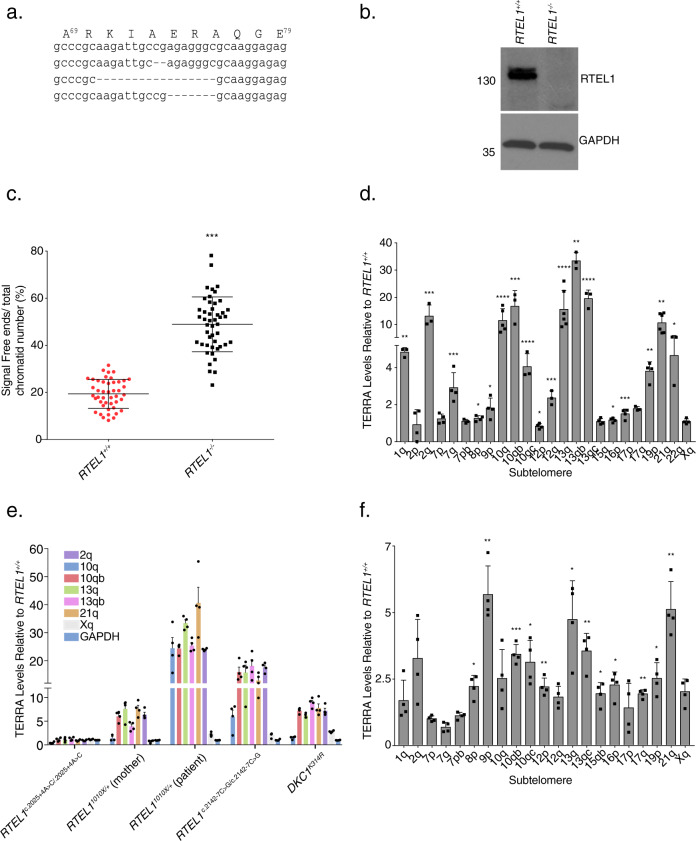
RTEL1 Influences TERRA Levels. **a** Sequence alignment of the PCR products amplified from the HEK293 cells genomic segment of *RTEL1* targeted by sgRNA revealing disruption of the coding sequence (exon #2 of *RTEL1)*. **b** Western blot of RTEL1 protein levels in wild type and *RTEL1* knockout HEK293 cells. Molecular weight markers, KDa are shown. Immunoblotting experiments were repeated at least three times producing similar results. **c** Quantification of telomere loss per chromatid. Data represents the average of at least 50 metaphases as mean and standard deviation (***p < 0.001, two-sided *t*-test). **d** TERRA levels at specific chromosome ends are elevated in RTEL1 deficient cells. Bars represent the relative TERRA expression measured by qRT-PCR at 18 chromosome ends. Values are sample averages of at least four experimental repeats (*p < 0.05, **p < 0.01, ***p < 0.001, ****p < 0.0001, two-sided *t-*test). **e** TERRA levels are elevated in cells from a panel of patient derived LCL lines. TERRA levels were measured by qRT-PCR. Values are sample averages of at least three experimental repeats and *p*-values are listed in Supplementary Fig. 9E (*p < 0.05, **p < 0.01, ***p < 0.001, ****p < 0.0001, two-sided *t*-test). **f** TERRA levels are elevated in a fibroblast cell line derived from a HHS patient homozygous for the *RTEL1*^*R1264H*^ allele. TERRA levels were measured by qRT-PCR. Values are sample averages of at least three experimental repeats (*p < 0.05, **p < 0.01, ***p < 0.001, ****p < 0.0001, two-sided *t*-test) (see also Supplementary Fig. 9). For qRT-PCR means and standard deviations are shown.

To understand the functional significance of RTEL1 binding to TERRA, we assessed the disposition of TERRA in the context of RTEL1 deficiency. *RTEL1* was inactivated via CRISPR-Cas9-mediated deletion of exon #2 in HEK293 cells (hereafter, RTEL1-KO)^55^ (Fig. 3a, b). The effect of RTEL1 deficiency on telomere stability was examined by fluorescence in situ hybridization (FISH) using a PNA-[TTAACCC]^3^ probe (Tel FISH). RTEL1-KO cells displayed a significant loss of telomere signal from one or both sister chromatids when compared to wild type HEK293 cells (48.9% ± 11.6 vs. 19.4% ± 6.2) (Fig. 3c), consistent with previous results in both human and mouse cells^1,6,15,56,57^. Similar results were obtained using a PNA-[TTAAGGG]^3^ probe (Supplementary Fig. 7C), indicating that the loss occurred on both strands of telomeric DNA.

We next examined the levels of TERRA RNA. TERRA transcription initiates at subtelomeric regions and extends into the telomeric repeats. The levels of TERRA transcripts originating at various chromosome ends was measured by quantitative reverse transcription PCR (qRT-PCR) using primers derived from the respective subtelomeric sequences^31,46,58^ (Supplementary Table 1). We tested 18 chromosome ends, some with more than one primer set. We found that TERRA levels were markedly elevated in RTEL1-KO cells when compared to wild type cells. The fold change was highest for chromosome ends 2q, 10q, 13q, and 21q (Fig. 3d). These results are consistent with the view that TERRA transcription is controlled by different factors at different chromosome ends^22,46^. RTEL1 deficiency did not affect the levels of mRNA transcribed from genes that are known to form R-loops^59^ or that of *NRAS* and *BCL2* mRNAs, the 5’ UTRs of which form G-quadruplexes that are bound by RTEL1 in vitro (Supplementary Fig. 6C). We examined the cell cycle profile for these cells to exclude the possibility of differences in cell cycle accounting for fluctuations in TERRA levels^60^ (Supplementary Fig. 8). Cycling cells were stained with propidium iodide for DNA content and analyzed by flow cytometry. The Dean-Jett-Fox algorithm FlowJo™ was used to calculate the percentage of cells in each phase of the cell cycle G0–G1 phase (purple), S phase (yellow) and G2–M phase (green). Wild type *RTEL1* cells have 44.1%, 34.2%, and 20.1% of cells in G1, S, and G2, respectively. RTEL-KO cells have a 30.3%, 33.7%, and 33.3% of cells in G1, S, and G2, respectively. These results suggest that cell cycle patterns alone do not account for TERRA fluctuations.

TERRA levels were also elevated in a panel of lymphoblastoid cell lines derived from HH and DC patients, as well as a fibroblast cell line homozygous for the *RTEL1*^*R1264H*^ mutation. The lymphoblastoid lines examined were derived from a patient with HH harboring an autosomal dominant mutation, *RTEL1*^*1010x/+*^ (NCI-164-1), the patient’s mother who presented with short telomeres but it is clinically unaffected (NCI-164-3), two cell lines from patients with HH due to with homozygous *RTEL1* splice variants (*RTEL1 c.2025*+*4A*>*C* (NCI-323-1) and *RTEL1 c.2142-7C*>*G* (NCI-347-1)), and a DC patient cell line with a dyskerin mutation (*DKC1*^*K314R*^ (NCI-106-3))^15,16,61^. Cells derived from a healthy donor proven negative for telomere biology gene mutations by exome sequencing (NCI-165-2) were used as the wild type control for lymphoblastoid cell lines, and a laboratory stock of previously immortalized BJ-hTERT’s were used as controls for *RTEL1*^*R1264H*^ fibroblast cell lines as no healthy donor samples were available.

TERRA transcripts emanating from the same subtelomeric regions as in the RTEL1-KO line (i.e., 2q, 10q, 13q, and 21q) were elevated in cells derived from the *RTEL1*^*1010x/+*^ proband, the proband’s mother, the *RTEL1 c.2142-7C*>*G*, and the *DKC1*^*K314R*^ patient samples (Fig. 3e and Supplementary Fig. 9C–E). TERRA levels were also significantly elevated in a fibroblast cell line derived from a HH patient homozygous for the *RTEL1*^*R1264H*^ allele^15^ (Fig. 3f). Conversely, TERRA levels were normal in the cells with the *RTEL1 c.2025*+*4A*>*C* mutation in which RTEL1 protein levels were not affected (Supplementary Fig. 9B), and TERRA transcripts emanating from chromosome 17p, which are regulated by CTCF binding^30^ were elevated in the *DKC1*^*K314R*^ and *RTEL1 c.2142-7C* > *G* cell lines (Supplementary Fig. 9D). 2q, 10q, 13q, and 21q transcripts were unaffected in that cell line. These data suggest that alterations in TERRA levels associated with defects in RTEL1 may contribute to the disease phenotypes in HH and in DC.

In our hands, the RTEL1-KO cell line exhibits limited growth capacity and becomes senescent within one to two months, consistent with previous data^62^. Complementation with *RTEL1*^1-762^, *RTEL1*^1-1118^, *RTEL1*^*∆762*^_,_ and *RTEL1*^*R1264H*^ (Fig. 1a), failed to rescue the senescence phenotype, suggesting that both the TERRA-binding domain and the helicase domain are required for viability. However, complementation with wild type *RTEL1* and the helicase deficient *RTEL1*^*K48R*^ mutant permitted the establishment of viable clones (Supplementary Fig. 9A), suggesting that helicase activity per se is not essential.

**Fig. 4:**
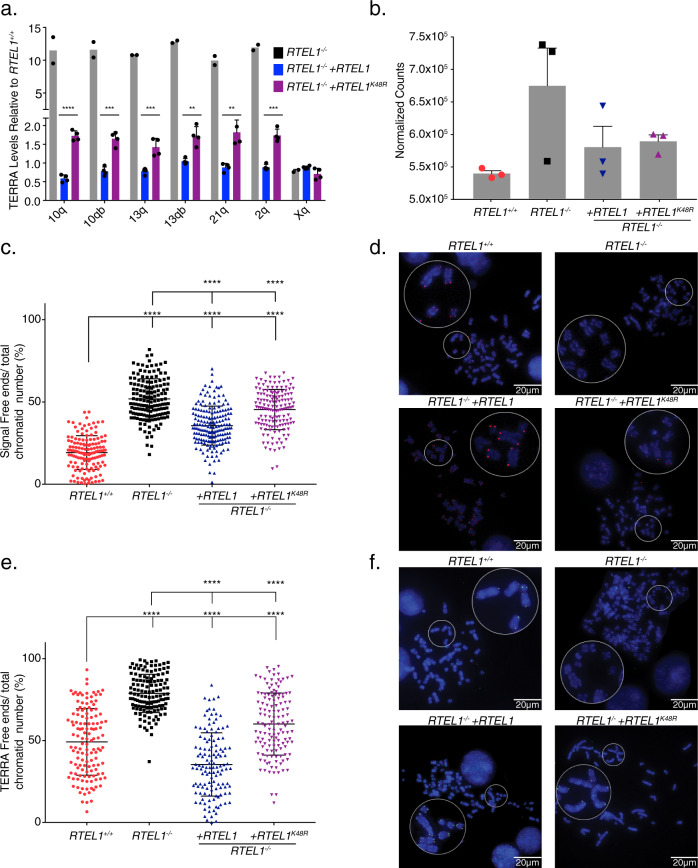
RTEL1 Influences TERRA Localization. TERRA levels are rescued upon RTEL1 complementation. **a** TERRA levels for four upregulated chromosome ends in the RTEL1-KO cell line were tested in cells complemented with *FLAG-RTEL1* and a catalytic site mutant *FLAG-RTEL1*^*K48R*^. Bars represent the relative TERRA expression measured by qRT-PCR in three independent experiments (*p < 0.05, **p < 0.01, ***p < 0.001, ****p < 0.0001, two-sided *t*-test) and mean and standard deviation is shown. **b** Total subtelomeric counts determined by RNA-seq experiments in the cell lines specified in **a**. Mean + /− SEM is shown. **c** Quantification of telomere loss per chromatid in *FLAG-RTEL1* and *FLAG-RTEL1*^*K48R*^ reconstituted cell lines by telomere FISH. **d** Representative telomere FISH images are shown with zoomed-in section (large white circle for zoom of small white circle). **e** Quantification of TERRA loss per chromatid in *FLAG-RTEL1* and *FLAG-RTEL1*^*K48R*^ reconstituted cell lines by TERRA FISH. **f** Representative TERRA FISH images are shown with zoomed-in section (large white circle for zoom of small white circle). Data for both TERRA and Telomere FISH experiments represent the average of at least 50 metaphases in each of three independent experiments and three independent reconstitutions shown as the mean and standard deviation (*p < 0.05, **p < 0.01, ***p < 0.001, ****p < 0.0001, two-sided *t*-test) (see also Supplementary Figs. 8 and 9).

Complementation of RTEL1-KO cells normalized TERRA levels in three independent transductions. Following introduction of wild type *FLAG-RTEL1*, TERRA levels decreased approximately ten-fold from all chromosomes tested (Fig. 4a and Supplementary Fig. 9A). TERRA levels were also reduced in *FLAG-RTEL1*^*K48R*^-expressing clones but remained significantly higher than cells complemented with wild type *RTEL1* (Fig. 4a). RNA-seq analyses of wild type, RTEL1-KO HEK293 cells and cells complemented with *FLAG-RTEL1* or *FLAG-RTEL1*^*K48R*^ showed that subtelomeric read counts in the RTEL1-KO were elevated and reduced upon complementation with *FLAG-RTEL1 or FLAG-RTEL1*^*K48R*^ (Fig. 4b). The magnitude of the increased read counts is less than observed in QPCR analyses, likely reflecting that TERRA increases were largely restricted to the four chromosome ends noted; RNA-seq represents an average high and low expression levels of TERRA coming from different chromosome ends. These results suggest that RTEL1 influences TERRA levels in a manner that is partially helicase dependent.

The normalization of TERRA levels in complemented cells was not correlated with restoration of telomeric DNA. We examined the levels of telomere loss in RTEL1-KO cells and cells complemented with *FLAG-RTEL1* and *FLAG-RTEL1*^*K48R*^ by Tel FISH. The frequency of telomeric signal free ends was only partially reduced in the reconstituted samples: 19%, 52%, 36%, and 45% for RTEL1 wild type, RTEL1-KO, *FLAG-RTEL1*, and *FLAG-RTEL1*^*K48R*^ reconstitutions, respectively (Fig. 4c, d). These data argue that increased TERRA levels are not simply a consequence of telomere shortening.

**Fig. 5:**
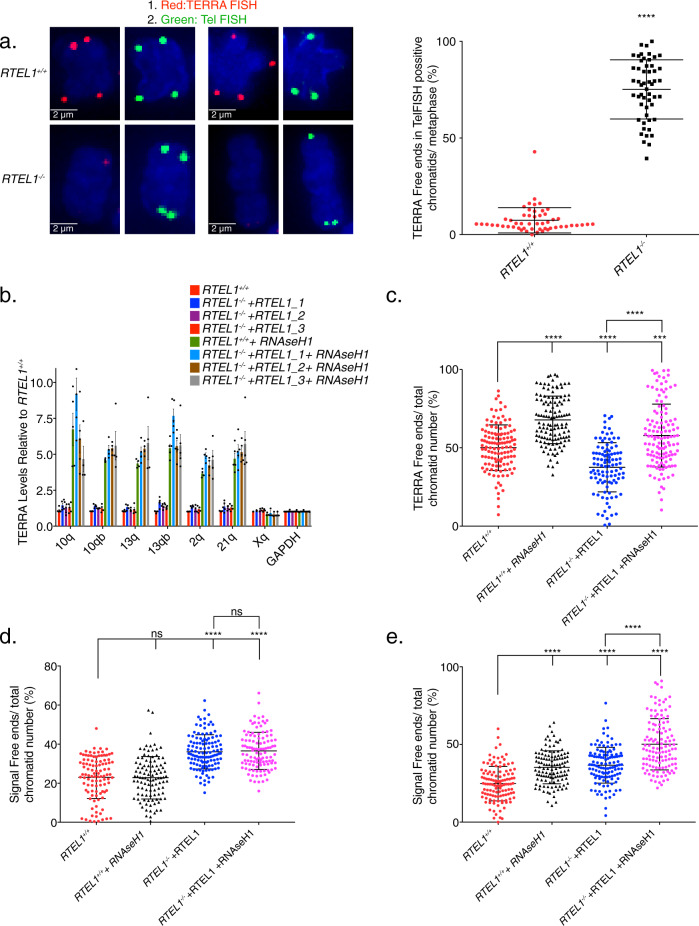
*RNAse H1* overexpression partially phenocopies RTEL1 deficiency. **a** Example chromosomes, and quantitation of TERRA free ends per chromatid in Tel FISH-positive chromatid ends. Samples were processed by sequential TERRA-Tel FISH and scored for TERRA (red) in telomeres marked by Tel FISH signal (green). Chromatid ends in at least 25 metaphases in two experiments were counted (****p < 0.0001, two-sided *t*-test). TERRA levels are elevated after RNAse H1 overexpression. **b** TERRA levels of specified chromosome ends were tested in cells transfected with *RNAse H1-GFP*. Bars represent the relative TERRA expression measured by qRT-PCR in three independent experiments *p*-values are listed in Supplementary Fig. 10B (*p < 0.05, **p < 0.01, ***p < 0.001, ****p < 0.0001, two-sided *t*-test). Means and standard deviation is shown. **c** Quantification of TERRA loss per chromatid in cells transfected with *RNAse H1-GFP* by TERRA FISH (22 days in selection). **d** Quantification of telomere loss per chromatid in cells transfected with *RNAse H1-GFP* by telomere FISH (22 days in selection). **e** Quantification of telomere loss per chromatid in cells transfected with *RNAse H1-GFP* by telomere FISH (52 days in selection). Three independent cell lines complemented with RTEL1 were averaged for analysis where specified. FISH experiments represent the average of at least 50 metaphases in each of 2 independent experiments and three independent reconstitutions shown as the mean and standard deviation (*p < 0.05, **p < 0.01, ***p < 0.001, ****p < 0.0001, two-sided *t*-test) (see also Supplementary Fig. 10).

To confirm that interpretation, we further examined the dependence of telomere length in TERRA R-loop formation using a sequential TERRA FISH-Tel FISH protocol^63^. Metaphase chromosomes from RTEL1 wild type and RTEL1-KO cells were subjected to two FISH staining steps. First, TERRA FISH was performed, then samples were destained, and conventional denaturing Tel FISH followed. Chromatid ends with clear Tel FISH signal were counted as “long telomeres” and scored for the corresponding TERRA signal in the same chromatids. The majority (75.2%) of long telomeres in the RTEL-KO cell line had TERRA free ends in comparison to 7.4% TERRA loss in the RTEL1 wild type cells (Fig. 5a). These data confirms that the increase in TERRA levels is not solely a consequence of telomere shortening, consistent with a role for RTEL1 in TERRA R-loop establishment and/or maintenance.

### RTEL1 influences TERRA localization

We next examined TERRA localization in RTEL1-KO cells. TERRA is localized to telomeric regions within R-loop structures that are established co-transcriptionally or post-transcriptionally by insertion of TERRA in trans^22,64^. To assess whether TERRA’s telomeric localization was affected by RTEL1 deficiency, we carried out RNA FISH for TERRA on metaphase spreads of RTEL-KO cells and cells complemented with *FLAG-RTEL1* and *FLAG-RTEL1*^*K48R*^ by TERRA FISH (Fig. 4e, f). Specificity of the probe was confirmed by treatment with RNAses where most of the TERRA signal was abolished upon treatment. About 10% of the signal was resistant to RNAse treatments (Supplementary Fig. 7D). The level of TERRA free telomeres in wild type cells was 49.2%, while the majority of the RTEL1-KO chromatids (78.8%) had no detectable TERRA signal. Complementation with wild type *RTEL1* reduced the number of TERRA free ends significantly, with the *FLAG-RTEL1* having a greater impact than *FLAG-RTEL1*^*K48R*^ (35.4% vs. 60% TERRA free ends).

Altogether, these data offer a different perspective on the role of RTEL1 and TERRA in stabilizing telomeric ends, suggesting that RTEL1 has a role in the establishment or stability of TERRA-containing R-loops while at the same time influencing the steady state levels of TERRA RNA.

These data raise the possibility of a relationship between TERRA R-loops and TERRA levels. Therefore, we targeted R-loops for destruction by overexpressing RNAse H1. *RTEL1* wild type cells and *RTEL-KO*-*FLAG-RTEL1-*complemented cells were transfected with *RNAse H1-GFP*. Following 12 days of G418 selection, the selected pool of cells was assayed for RNAse H1-GFP expression (Supplementary Fig. 10A), and the levels of and localization of TERRA were assessed. We found that TERRA levels in the RNAse H1 expressing lines were significantly elevated, thus partially phenocopying *RTEL1* deficiency (Figs. 5b, 3d, and Supplementary Fig. 10B). This outcome was not related to differences in cell cycle progression. Wild type (parental) and RTEL-KO cells reconstituted with wild type *RTEL1* expressing *RNAseH1* did not exhibit significant differences in growth profiles (Supplementary Fig. 8). These data argue that TERRA R-loops influence TERRA expression in wild-type cells.

As expected, the telomeric localization of TERRA, which is R-loop dependent, was also diminished after *RNAse H1-GFP* overexpression (Fig. 5c). The level of TERRA free ends in the wild type cells were 50%, while 68% of wild type RTEL1 cells with *RNAse H1-GFP* had no TERRA signal. Similarly, TERRA free ends were increased from 38% to 58% in *FLAG-RTEL1* complemented RTEL-KO cells in which *RNAse H1-GFP* was overexpressed (Fig. 5c).

The frequency of telomeric signal free ends at 22 days post transfection was not significantly affected by *RNAse H1-GFP* overexpression: 23%, 23%, 36%, and 37% for RTEL1 wild type, *RTEL1* wild type + *RNAse H1-GFP*, *RTEL-KO-FLAG-RTEL1* and *RTEL-KO-FLAG-RTEL1*+*RNAse H1-GFP*, respectively (Fig. 5d). However, at 52 days, we observed a marked increase in the frequency of signal free telomeric ends; 24.8% *vs*. 35.3% for RTEL1 wild type compared to RTEL1 wild type+*RNAse H1-GFP* and 36.7%, vs. 50.2% in *RTEL-KO-FLAG-RTEL1* and *RTEL-KO-FLAG-RTEL1*+*RNAse H1-GFP*. In contrast, no significant difference in TERRA levels was observed at this time point (Fig. 5e and Supplementary Fig. 10C).

## Discussion

In this study, we established that RTEL1 influences the abundance and localization of the telomeric RNA TERRA, likely through direct binding to TERRA via a previously undescribed domain at the RTEL1 C-terminus. In vitro analyses revealed that RTEL1 binds G-quadruplex DNA and RNA, but the differential affinity for folded vs. unfolded RNA is significantly higher than for the analogous structures in DNA. In cells, *RTEL1* hypomorphism and deficiency are associated with telomere fragility and loss, as noted previously^1,6,15,56,57^. However, we observed a marked increase in TERRA levels in *RTEL1* deficient cells. Increased levels of TERRA was not associated with increased telomeric localization. Rather, in *RTEL1* deficient cells, the frequency of TERRA occupancy at the telomeres of metaphase chromosomes was markedly reduced.

Reconstitution of *RTEL-KO* cells revealed that the effects on TERRA were only partially helicase dependent. We also found that *RTEL1* cDNAs encoding RTEL1 variants that lacked the G4-binding domain were unable to support clonal survival of the *RTEL1* knockout line, indicating the physiological importance of G4 engagement by RTEL1. The observation of elevated TERRA levels in *RTEL1* knockout cells was recapitulated in several lymphoblastoid cell lines from HH and DC patients, as well as in a fibroblast line derived from the HH patient homozygous for the *RTEL1*^*R1264H*^ allele^15,16^. In each of those cases, *RTEL1*^*1010X*^*, RTEL1*^*R1264H*^, and the *RTEL1*^*c.2142-7C>G*^ mutations predicted to alter splicing^61^, the RTEL1 C-terminus is affected (Figs. 1a and 3e, f). These data raise the possibility that telomere dysfunction may be caused by alterations of TERRA abundance and localization associated with RTEL1 deficiency, and this may contribute to the clinical phenotypes of DC and HH.

In addition to binding TERRA, the RTEL1 C-terminal domain promotes oligomerization via the C4 domain (Fig. 1c). In this regard, it is notable that some *RTEL1* alleles behave as autosomal dominant mutations with respect to clinical presentation (*RTEL1*^*1010X*^) or sub-clinically with respect to telomere length; the *RTEL1*^*R1264H*^ heterozygous parents of homozygous *RTEL1*^*R1264H*^ patients exhibit pronounced telomere shortening^15,16,61^. Supporting the importance of oligomerization, overexpression of *RTEL1*^*R1264H*^ in wild type HEK293 cells leads to rapid selection against *RTEL1*^*R1264H*^ expressing cells, whereas overexpression of *RTEL1* cDNAs that encode proteins lacking the RING domain was well tolerated. Since the RTEL1 helicase domain is an independent oligomerization interface, the apparent toxicity of the RTEL1^R1264H^ may reflect that RTEL1 assemblies in which the C-terminal homotypic interaction is impaired are toxic. The RING domain specifies functions aside from dimerization. For example, the interaction of RTEL1 with TRF2 is lost in the *RTEL1*^*R1264H*^ mutant^65^; hence the apparent toxicity of *RTEL1*^*R1264H*^ may not be due to an effect on dimerization. Nevertheless, these data support a model where both oligomerization and RNA binding are important for RTEL1 function independent of its helicase activity.

**Fig. 6:**
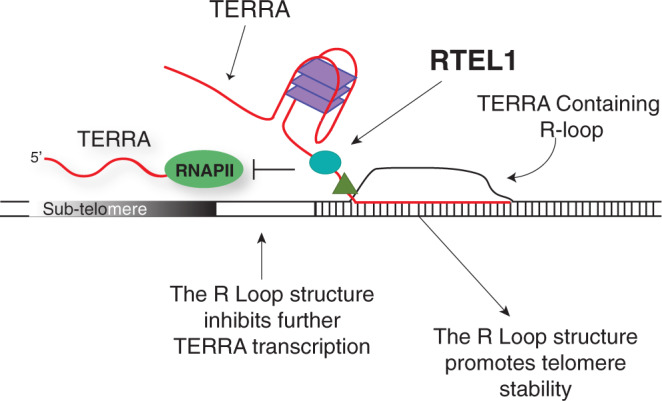
RTEL1 Influences the Abundance and Localization of the Telomeric RNA TERRA. The formation and maintenance of the TERRA R-loop at the telomere influences TERRA transcription and promotes telomere stability. We propose RTEL1 would be involved in either facilitating the creation or contributing to the maintenance of the TERRA-containing telomeric R-loop. Once established, the TERRA R-loop would limit further transcription from R-loop containing chromosome ends.

On the basis of the data obtained, we propose that the formation and maintenance of the TERRA R-loop at the telomere influences TERRA transcription and promotes telomere stability. In this conception, RTEL1 would be involved in either facilitating the creation of the TERRA-containing telomeric R-loop or contributing to its maintenance. Once established, the TERRA R-loop would limit further transcription from R-loop containing chromosome ends (Fig. 6). This part of the model is based on the reduction in TERRA R-loops and increased levels of TERRA in RTEL1 deficient cells (Figs. 3c–f and 4e, f). It is likely that the ability of RTEL1 to bind and discriminate distinct nucleic acid folds in vitro is biologically relevant at the telomere where these structures are predicted to form. Implicitly, the model also predicts that the presence of the R-loop at the telomere contributes to telomere stability.

The phenotypic outcomes of *RNAse H1* overexpression, which is known to disrupt R-loops^43^, were consistent with the model proposed. First, *RNAse H1* overexpression increased the frequency of metaphase chromosomes that lacked TERRA FISH signal. This apparent reduction in TERRA R-loops was associated with increased levels of TERRA RNA, as inferred by Q-PCR. Notably, *RNAse H1* overexpression increased TERRA levels from chromosomes 2q, 10q, 13q, and 21q (Fig. 5b), but not chromosomes 8p, 19p, or Xq (Fig. 5b). Transcription of TERRA from those same chromosomes also increases upon *RTEL1* inactivation and in hypomorphic patient cells (Fig. 3d–f and Supplementary Fig. 9C, D), strengthening the idea that the R-loop exerts an influence on TERRA abundance, at least from those telomeric loci.

Second, the model predicts that the TERRA R-loop would contribute to telomere stability, and accordingly that *RNAse H1* overexpression would decrease telomere stability. At 12 and 22 days after transfection of *RNAse H1*, R-loops, as inferred from TERRA RNA FISH, were diminished but no increase in telomere-free chromatid ends was evident, consistent with previous data (ref. ^43^ and Fig. 5c, d). As noted above, TERRA levels were elevated at those time points. At 52 days post transfection, a significant increase in telomere loss was evident (Fig. 5e), again consistent with the model.

However, at the 52 day time point, TERRA levels were no longer elevated. On its face, this observation is inconsistent with the simplest interpretation of the model, but it is conceivable that prolonged *RNAse H1* overexpression may exert an indirect effect on TERRA expression. R-loops have been shown to prevent methylation of promoters by excluding binding of a methyltransferase required for proper gene silencing^66^. Some TERRA promoters are regulated by DNA methylation and depletion of, or defects in methyltransferases lead to elevated TERRA levels^22,31,67^. TERRA also interacts with ATRX, and antagonizes its functions^24^. Hence it is also conceivable that prolonged absence of TERRA-containing R-loops allows ATRX-mediated silencing of TERRA promoters. TERRA expression may also be reduced in an R-loop independent manner.

The role of RTEL1 in regulating R-loops is likely to be complex, and may involve both negative and positive regulation of those structures, likely through its binding to G4 structures. R-loops are associated with GC rich genomic contexts^59^ and recent data suggest that RTEL1 dismantles R-loops at loci in which G-quadruplex forming sequences are common, including those within common fragile sites^10^.

On the other hand, there is precedent for G4-binding helicases promoting the post-transcriptional formation of R-loop structures. For example, immunoglobulin class switch regions are G-rich repetitive elements that must be transcribed in order for class switch recombination to occur. Debranching of the spliced switch region transcript and its subsequent insertion into an R-loop structure requires the action of DDX1, an RNA helicase that binds the G4 structure of the transcript and inserts the RNA into the switch region DNA duplex to form the R-loop structure required for class switch recombination^68,69^. In addition, it is clear that TERRA can be inserted into R-loop structures in trans^24,29,64^, although the helicase required and whether it depends on G4 structure is not known.

The model proposed here wherein RTEL1 promotes telomere stability via its influence on the localization and abundance of TERRA RNA does not exclude other models to explain the role of RTEL1 in stabilizing the telomere. A prevalent model suggests that RTEL1 disassembles T-loops to allow DNA replication to proceed to the chromosome end^6,56^. When RTEL1 is depleted the T-loops are persistent and inappropriately resolved by the SLX4 nuclease. Loss of RTEL1-dependent resolution of the T-loop or other secondary structures has also been suggested to promote replication fork reversal upstream of the secondary structure obstacle. This pathological situation is proposed to be stabilized by binding of the TERT protein to the reversed fork and again lead to cleavage by SLX4^5^.

These models account for the detection of extrachromosomal telomeric circles, and for telomere fragility. A prediction of these models is that telomere loss should be identical on both sister chromatids, since the SLX4 cleavage event occurs before replication. Based on telomere FISH, this is true for a subset of chromosomes, but loss of telomeric signal on one sister chromatid is often observed (refs. ^6,15,56^ and Fig. 4d herein). This signal heterogeneity suggests that there may be more than one mechanism for how the telomeres are maintained by RTEL1, with some loss of telomeric sequences arising via the model proposed here (Fig. 6). Additionally, a new report suggests that rapid telomere deletion in the form of T-circles may not cause telomere shortening in an RTEL1 deficient setting^70^. T-circle formation varied according to telomerase status, cell type and RTEL1 function^70^.

Recruitment of TERRA to short telomeres has been described in yeast and mammalian cells where it is suggested to help maintain telomeres by promoting recombination or recruitment of telomere maintenance factors like telomerase^41,44,64^. Telomeres are proposed to have either the ability to retain more TERRA when they are short or have active removal of TERRA RNA in a setting with long telomeres^64^. Furthermore a RAD51-mediated strand-invasion-like reaction was shown to be required for the insertion of TERRA R-loops into the telomere repeats and the resulting R-loops are sensitive to *RNAse H1*^64^. The mechanistic steps and totality of factors that are involved in forming and maintaining the TERRA R-loop remain unknown.

The data presented herein suggest a mechanism to account for RTEL1’s influence on telomere stability, and may illuminate the biological significance of RTEL1–TERRA interaction. The data further support the view that TERRA plays a role in stabilizing telomeric sequences.

## Methods

### Cell lines

HEK293 and HEK293T cells were grown in 10% DMEM and 10% FBS. EBV-immortalized lymphoblastoid cell lines were derived from participants in the National Cancer Institute’s IRB-approved longitudinal cohort study entitled “Etiologic Investigation of Cancer Susceptibility in Inherited Bone Marrow Failure Syndromes” (ClinicalTrials.gov Identifier: NCT00027274)^71^. This study includes comprehensive family history and individual history questionnaires, detailed medical record review, and biospecimen collection. Detailed clinical evaluations were performed at the NIH Clinical Center per protocol. LCLs were grown in RPMI Media supplemented with 100 U/ml penicillin, 100 μg/ml streptomycin, GlutaMAX (Life Technologies), and 20% FBS. hTERT transduced fibroblasts were cultured in DMEM media supplemented with 100 U/ml penicillin, 100 μg/ml streptomycin, GlutaMAX, and 15% FBS. All cell lines used were routinely checked for mycoplasma.

### Protein expression and purification

Sequences encoding residues 762-1300, 762-1097, 1097-1300, 1118-1300, and 1143-1300 of human RTEL1 were amplified by PCR then subcloned between the *EcoRI* and *XhoI* restriction sites of *pET28* (Novagen). RTEL1 proteins were expressed as 6-histidine-fusions in the *E. coli* strain BL21 and purified using His-trap (GE Healthcare), Hi-Trap Q (GE Healthcare), and Superdex 200 columns (GE Healthcare). For expression in mammalian cells, full-length human *RTEL1* and *RTEL1*^1-1097^ were subcloned in to *pcDNA3.1 MCS-BirA (R118G)-HA* between *HpaI* and *EcoR1* sites. *pcDNA3.1 MCS-BirA(R118G)-HA* was a gift from Kyle Roux (Addgene plasmid # 36047). Full-length *RTEL1* was cloned into *pDest12.2-myc-his* and *pDESTb-FRT-C3xFLAG*. RTEL1^∆1097^ was amplified by PCR and subcloned between *EcoRI* and *XhoI* restriction sites of *pCDNA3.1_FLAG-HA* (Invitrogen) and *pIC113* (Addgene plasmid # 44434). For expression of RTEL1 proteins in HEK293 RTEL1-KO cells, full-length human *RTEL1* was cloned into *pLenti-C-myc-DDK-IRES-Puro* (Origene) plasmid by digestion with *AscI* and *MIuI* and mutagenesis for *RTEL1*^*K48R*^ was performed using QuikChange Lightning Multi Site-Directed Mutagenesis Kit (Agilent). Cells were infected with lentiviruses expressing the indicated RTEL1 proteins and infected cells were selected with puromycin as previously described^72^. Cell lines derived from three independent infections were used for reconstitution experiments. The *pEGFP‐RNAse H1* was a gift from Andrew Jackson & Martin Reijns (Addgene plasmid # 108699). HEK293 were transfected using PEI-25K (polysciences Inc.) and after 48 h selected with G418 for the indicated time points. For RNA-IP experiments FLAG-RTEL1, FLAG-∆762 were cloned into pCMV6-AC-DDK (Origene) plasmid and FLAG-TRF1 was a gift from Titia de Lange (Addgene plasmid # 16058).

### Co-immunoprecipitation and western blotting

HEK293T cells were transfected with wild type and single point mutants of the following constructs as indicated: *RTEL1-myc-HIS*, *RTEL1-FLAG*, *GFP-RTEL1*^*∆1097*^, and *FLAG-HA RTEL1*^*∆1097*^ using PEI-25K (polysciences Inc.). An additional nuclear localization signal (MADPKKKRK) was added to the 5’ end of the *RTEL1* truncation constructs and mutagenesis was performed using standard protocols. Cell extracts were prepared using 1X PBS, 0.5% (v/v) Triton-X-100 and protease inhibitor tablet (Sigma). Proteins were immunoprecipitated by incubating cell extracts overnight with either FLAG M2 (Sigma) or GFP (Clonetech) antibodies at 4 °C, followed by incubation with Protein A/G beads for 3 h. Lentiviral transfections were performed as previously described^52^. Western blotting was performed using standard methods. Total extracts were prepared in lysis buffer (60 mM Tris-HCl pH 6.8, 2% SDS) and analyzed with specified antibodies. The antibodies used in this study were GFP JL-8 (Clonetech, 632381, 1:4000), ANTI-FLAG® M2 (Sigma, F1804, 1:1000), HA (Biolegend #901515, 1:1000), myc 9E10 (BioXcell, BE0238, 1:4000), RTEL1 (custom made 1:2000–5000), Actin-HRP (ABCAM, ab49900 1:50,000), GAPDH (Santacruz, sc_32233, 1:1000) and RNAse H1 (Proteintech 15606-1-AP, 1:1000).

### Size exclusion chromatography coupled to multi-angle light scattering (SEC-MALS)

The molar masses of RTEL1 proteins were analyzed using SEC-MALS. In all, 200–300 µg of the indicated proteins were injected into a Superdex 200 10/300 GL (GE Healthcare) equilibrated at a flow rate of 0.3 ml per minute in 50 mM Hepes pH 7.5, 1 mM TCEP, and either 150 or 300 mM NaCl. Light scattering was monitored with a miniDAWNTREOS system (Wyatt Technology), concentration was measured with the Optilab T-rEX differential refractometer (Wyatt Technology), and molar masses were calculated using the Astra 6.1 program (Wyatt Technology) with a d*n*/d*c* value of 0.185 ml/g.

### BioID

Affinity purification of biotinylated proteins was performed as previously described^47^. Briefly cells were supplemented with 50 µM biotin for 24 h and sonicated in lysis buffer containing 50 mM Tris, pH 7.4, 500 mM NaCl, 0.4% SDS, 5 mM EDTA, 1 mM DTT, 1x Complete protease inhibitor (Roche). Triton-X-100 was brought up to 2% and an equal volume of 50 mM Tris, pH 7.4 was added before clarifying the lysate by centrifugation. Supernatants were incubated with 200 µl Dynabeads (MyOne Steptavadin C1; Invitrogen) overnight. The beads were washed with 1.5 ml wash buffer 1 (2% SDS), 1.5 wash buffer 2 (0.1% deoxycholate, 1% Triton-X-100, 500 mM NaCl, 1 mM EDTA, and 50 mM Hepes, pH 7.5), 1.5 ml wash buffer 3 (250 mM LiCl, 0.5% NP-40, 0.5% deoxycholate, 1 mM EDTA, and 10 mM Tris, pH 8.1). After the wash 1.5 ml (50 mM Tris, pH 7.4, and 50 mM NaCl) and 50 µl 50 mM NH_4_HCO_3_ were added before mass spectrometry analysis. On- bead tryptic digestions were analyzed by the MSKCC Proteomics Core as previously described^47^. MassHunter Qualitative Analysis software (Agilent Technologies) was used to analyze the raw data and database search was done using MASCOT (MatrixScience). SCAFFOLD Q + (Proteome Software) was used to visualize the data with a threshold of at least 2 identified peptides and a minimum 95% probability each.

### Binding assays

Oligonucleotides were refolded in 10 mM Tris pH 7.5 and 100 mM KCL (unless otherwise noted) with slow cooling starting at 95 °C down to room temperature. The change in fluorescence anisotropy of the indicated 5’ fluorescein labeled oligonucleotides was measured to determine the relative binding affinities of RTEL1 proteins. The labeled oligonucleotides were mixed in 20 μl reaction volumes with the indicated RTEL1 proteins at concentrations ranging from 0 to 5 μM (unless otherwise indicated) in a reaction buffer containing 20 mM Tris, pH 7.5, 50 mM KCl, 0.5 mM TCEP, 0.5 mM MgCl_2_, 10% glycerol, and 0.1% IGEPAL in a 384-well microplate. The binding data were collected on a SpectraMax M5 (Molecular Devices) using a 495 nm excitation wavelength and a 525 nm emission wavelength. Apparent *K*_D_ values were calculated from triplicate experiments using a model for receptor depletion and plotted using Prism 7 GraphPad Software. The model used was: *Y* = Af + (Ab − Af)*((*L* + Kd + *X*) − sqrt((sqr( − *L* − Kd − *X*)) − 4*L*X))/(2*L). Where *A* is the anisotropy measured; *A*_b_ is the anisotropy at saturation (100%), *A*_f_ is the anisotropy of the free oligonucleotide, *L* is the fixed concentration of the oligonucleotide and *X* is the protein concentration. For competition assays, RTEL1-FAM-TERRA-MUT (wild type TERRA RNA was not readily competed by any of the oligonucleotides tested) complex concentration was maintained at 200 nM or 1 µM as indicated. Competitor oligonucleotides were added at concentrations ranging from 0 to 20 µM as specified. Experiments were performed in triplicate, and the data were fit to a one-site-binding model for competition assays using Prism 7. For EMSA (Electrophoretic mobility shift assay), binding reactions were performed in buffer containing 20 mM Tris, pH 7.5, 50 mM KCl, 0.5 mM TCEP, 0.5 mM MgCl_2_, 10% glycerol, and 0.1% IGEPAL. RTEL1 proteins and 5’ FAM- labeled oligonucleotide at the indicated concentrations were incubated at room temperature for 30 min then loaded onto a 4–20% polyacrylamide non-denaturing gel. The gels were imaged using fluorescein fluorescence on a Typhoon FLA9500 instrument (GE).

### Quantitative reverse transcription PCR (RT-qPCR)

Total RNA was extracted from the specified cell lines using the RNeasy Mini Kit (Qiagen) with DNase treatment. cDNA was synthesized using Superscript IV kit (Invitrogen) using random primers. TERRA expression in each sample was normalized to *GAPDH* and relative expression was determined with the comparative CT method. Primers used in this study are listed in Supplementary Table 1.

### DNA and RNA FISH

Metaphase chromosome spreads were prepared by incubating cells with 0.1 µg/µl KaryoMAX colcemid solution for 90 min. Cells were harvested at 1000 rpm and resuspended in 0.075 M KCL at 37 °C for 15 min. Cells were fixed in a 3:1 mixture of ice-cold methanol/acetic acid at least overnight and spread onto glass slides then air dried overnight. Samples were treated with 100 µg/ml RNAse A (Sigma) for 1 h at 37 °C, dehydrated through a series of ethanol washes at 70%, 90%, and 100% ethanol for 5 min each at room temperature, then allowed to air dry. Hybridization was in buffer containing 10 mM Tris pH 7.5, 70% formamide, 0.5% blocking reagent (Roche) and 0.5 µg/ml CY-3 (CCCTAA)_3_ or Alexa 488 (GGGTAA)_3_ (PNA BIO), denatured at 75 °C for 5 min and incubated at room temperature for 16 h. Slides were washed twice in buffer containing 10 mM Tris pH 7.5, 0.1% BSA, and 70% formamide, then three times in PBS/0.15% Triton-X-100. The slides were mounted using proLong Gold antifade reagent with DAPI (Invitrogen). For TERRA FISH, slides were treated with either 100U RNAse H1 (NEB) alone or a mixture of RNAse A and RNAse H1 in PBS and 1x RNAseH1 buffer for specificity controls, or in buffer without RNAses for at least 6 h then treated as above with the exception of the denaturation step. Sequential FISH was performed as previously described^63^. Briefly, metaphase spreads were first stained by TERRA FISH. Images were collected and coordinates were recorded for selected metaphases. Samples were subsequently destained by washing with PBS then 2x SSC. The same slides were then processed for Tel FISH and images of the pre- recorded metaphases were captured. Metaphases were scored for Tel FISH and TERRA FISH to determine TERRA loss at chromatid ends with clear Tel FISH signal. Images were acquired on a Deltavision Imaging Elite System (GE Healthcare) with a CMOS Camera on an Olympus IX-71 microscope using a 60x objective. Images were analyzed using ImageJ and statistical analysis was done with Prisim 7 (Graphpad).

### Generation of the *RTEL1* knockout line

RTEL1 knockout was performed using CRISPR-Cas9-mediated genome editing as described previously^55^. We used a guide RNA mapping to Exon 2 of the *RTEL1* gene 5′-TGCCCGCAAGATTGCCGAGA. PCR-genotyping was performed using the following primers: 5′- GGGACTTGCCTGTGGACTTCTCCGC and 5′-CGCCATCCCTTGCCAACCATCCCC. PCR products were cloned using the Zero Blunt PCR Cloning Kit (Thermo Fisher Scientific, K270040) and 20 clones were sequenced per cell line to verify successful genome editing. Cells were grown from single cell clones until enough material was available for experiments ~2.5+ months.

### RNA crosslinking and RNA-IP

To analyze the RNA crosslinking capacity of wild type and mutant RTEL1 proteins, RTEL1-KO cells were complemented with N-terminally FLAG-HA-tagged wild type or mutant RTEL1. Cells were fed with 4-thiouridine (100 M) for 16 h and crosslinked at 365 nm. A modified version of PAR-CLIP^52^ was performed using a single RNase A digestion step and using anti-FLAG-M2 magnetic beads (Sigma, M8823) for IP.

RNA immunoprecipitation, RNA purification, DNAse treatment, RNAse treatment and slot blotting was performed as previously described^53^ with the following modifications: HEK293T cells were transfected with FLAG-RTEL1, FLAG-∆762 and FLAG-TRF1 using PEI-25K (polysciences Inc.). Flag M2 beads (Sigma) were pre-incubated overnight with 1% BSA in PBS before IP. TERRA probe containing 800 bp TTAGGG repeats was obtained by *Not*I/SacII restriction digest and *pSXneo 135(T2AG3)* Addgene (#12402). A 554 bp long *cMYC* probe was generated by *Bam*HI/*Sac*II digest of *pCDNA3- cMYC* (addgene #16011). Probes were random prime radiolabeled (α32P-dCTP) using the Amersham Megaprime DNA Labeling System (cytiva) following the manufacturers instructions. After UV crosslinking and baking the membrane at 65 °C for 1 h, the membrane was prehybridized at 50 °C for 1 h in 10 ml ULTRAhyb buffer (Thermo Fisher Scientific). The probes were denatured for 5 min at 95 °C prior addition to the hybridization tubes. Following hybridization over night at 50 °C, the membranes were washed five times for 30 min with 2xSSC and 0.1% SDS at 50 °C. The images were scanned followed by quantification with ImageGauge software (GE Healthcare).

### RNA-seq analysis for subtelomere counts

RNAseq libraries were prepared from total RNA using ribosomal RNA depletion method. Paired-end reads were aligned using STAR against subtelomeric sequences (subtelomeric sequences cover 15 kb before the start of the telomeric sequences TTAGGG for 1p,1q,2p,2q,3p,3q,4p,4q,5p,5q,6p,6q,7p,7q,8p,8q,9p,9q,10p,10q,11q,12p, 12q,13q,14q,15q,16p,16q,17p,17q,18p,18q,19p,19q,10p,10q,21q,22q,Xp,Xq.Yq ends and were provided by Dr. Joachim Linger). Mapped reads were counted using Featurecounts, allowing multi-mapping reads, and counting each alignment fractionally. The raw total counts of all the subtelomeric sequences for each sample were normalized using size factors from DESeq2. The size factors were generated from the whole transcriptome analysis for the same samples.

### Flow cytometry

Cell cycle analysis was performed by flow cytometry. Cells were fixed, RNase treated, and stained with propidium iodide. Data were analyzed with FlowJo™ software and percentages of cells in different stages of the cell cycle were calculated using the Dean-Jet-Fox model. G0–G1 phase (purple), S phase (yellow) and G2–M phase (green). Percentages of each phase are listed below each plot.

### Statistics

At least three independent experiments were carried out for each data set unless otherwise stated. Error bars, the number of replicates and *p*-values are shown in the figures and legends. All data was represented and statistically analyzed with Prism7 using two-tailed Student’s *t*-tests where indicated.

### Reporting summary

Further information on research design is available in the Nature Research Reporting Summary linked to this article.

## Supplementary information

Supplementary Information

Reporting Summary

## Data Availability

Data from this study are available from the corresponding author upon reasonable request. Source data are provided with this paper.

## Source data

Source Data
