# Supplementary material for: RTEL1 influences the abundance and localization of TERRA RNA
Source: Nat Commun. 2021 May 21;12:3016. doi: 10.1038/s41467-021-23299-2 (PMC8140157; doi:10.1038/s41467-021-23299-2)
Supplement: Supplementary file 1 — Supplementary Information [file 41467_2021_23299_MOESM1_ESM.pdf]

Supplementary Figure 1

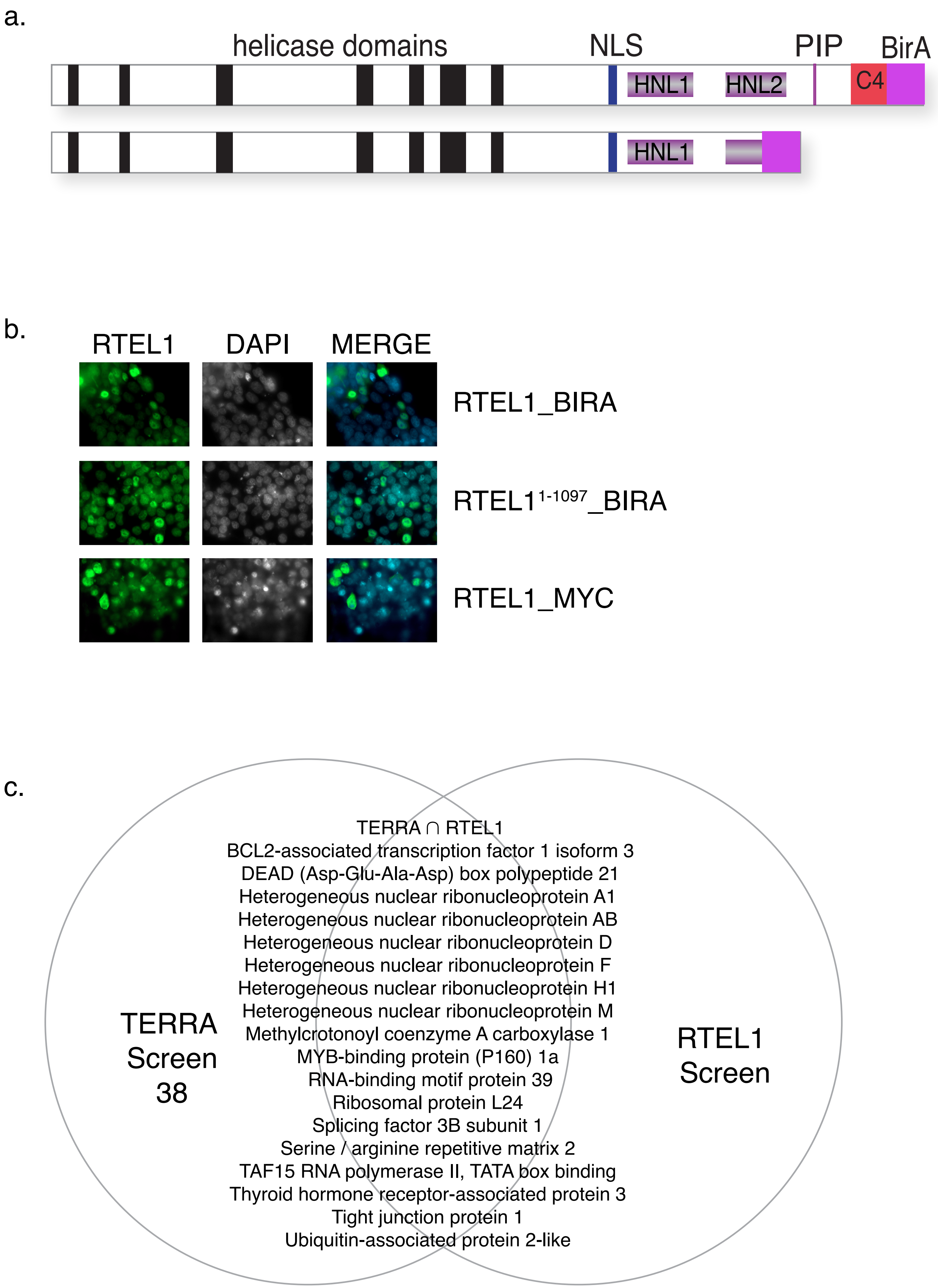

Supplementary Figure 1

(A) Schematic representation of *RTEL1-BirA* constructs used in this study. (B) RTEL1- BirA fusions did not affect the primarily nuclear localization of RTEL1-BirA compared to myc-tagged RTEL1. (C) Close to 50% of proteins obtained in a Biotin-TERRA pull-down (Lopez de Silanes, Stagno d'Alcontres et al. 2010) matched proteins that were enriched for in the *RTEL1-BirA* construct.

Supplementary Figure 2

a.

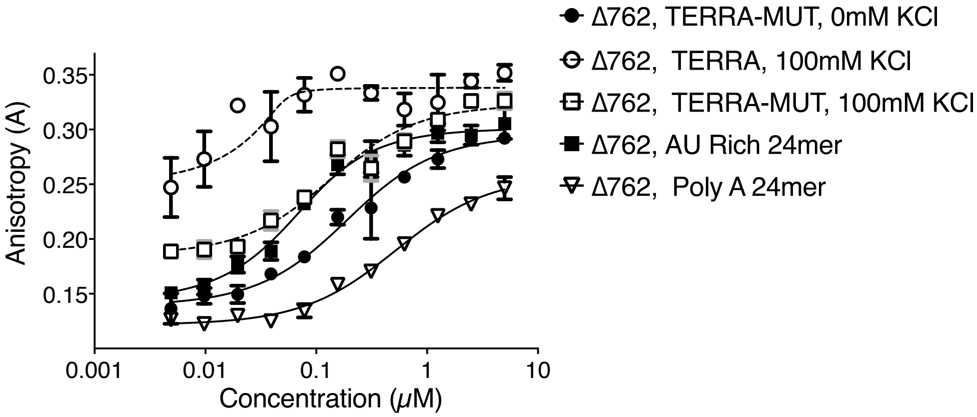

Oligo	KD (nM)
AU Rich 24mer	42.0 ± 6.1
Poly A, 24mer	497.6 ± 47.8
TERRA, 0 mM KCl	37.1 ± 9.6
TERRA-MUT, 100 mM KCl	107.7 ± 21.6
TERRA, 100 mM KCl	1.3 ± 3.6

b.

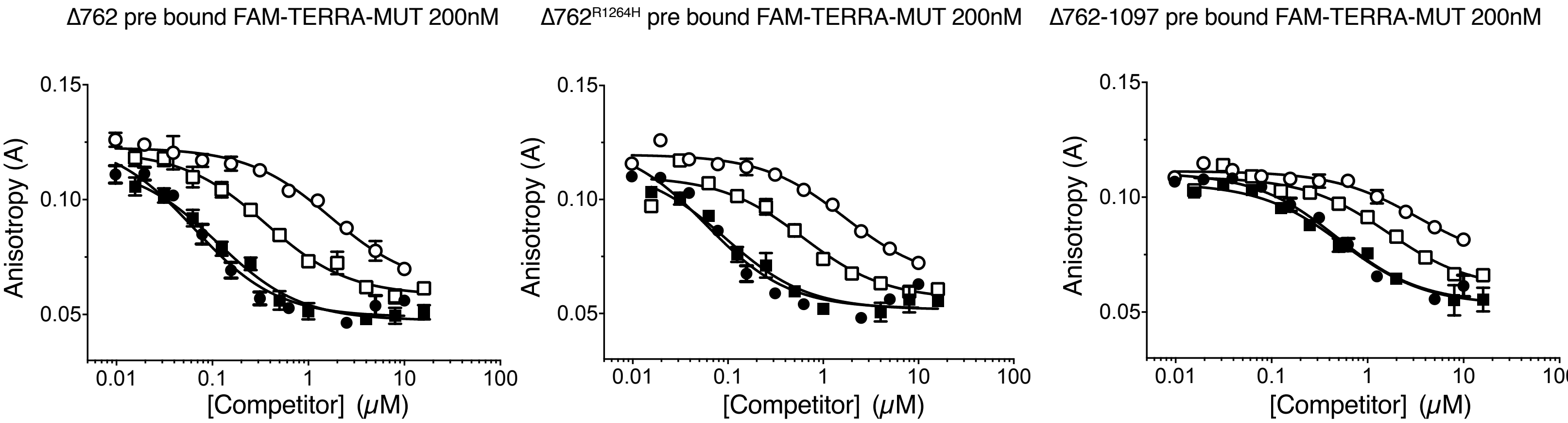

- TERRA-competitor
- TERRA-MUT-competitor
- hTelo-competitor
- hTelo-MUT-competitor

	TERRA	TERRA-MUT	hTelo	hTelo-MUT
	Ki (uM)	Ki (uM)	Ki (uM)	Ki (uM)
Δ762	0.017 ± 0.003	0.442 ± 0.074	0.029 ± 0.004	0.093 ± 0.011
$\Delta762^{R1264H}$	0.030 ± 0.007	0.850 ± 0.125	0.047 ± 0.009	0.309 ± 0.068
Δ762-1097	0.245 ± 0.035	1.652 ±0 .377	0.276 ± 0.039	0.826 ± 0.134

Supplementary Figure 2

Experimental fluorescence anisotropy data used to calculate apparent dissociation constants for RTEL1 proteins binding to RNA and DNA oligonucleotides indicated in Fig. 2. (A) Binding curves for TERRA and TERRA-MUT RNAs folded in the presence and absence of KCl, and AU-rich and polyA RNA controls. (B) Increasing concentrations of the indicated oligonucleotides were added to reactions containing RTEL1 proteins and a 24mer FAM-TERRA-MUT RNA at 200 nM. Curves for the bound FAM-TERRA-MUT competed by the indicated oligonucleotides are shown. All binding assays were conducted in triplicate and mean and standard deviation are shown.

Supplementary Figure 3

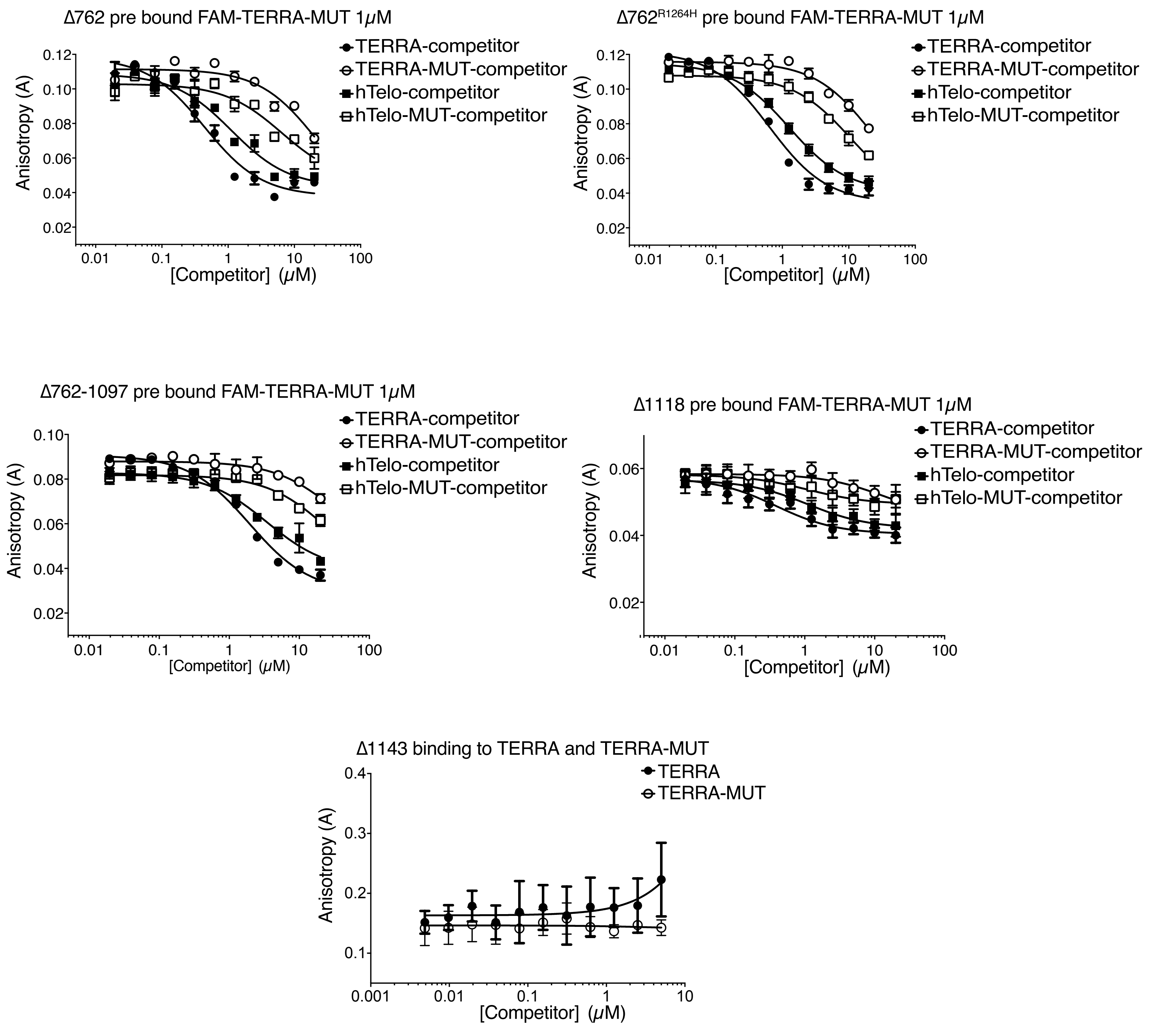

	TERRA	TERRA-MUT	hTelo	hTelo-MUT
	Ki (uM)	Ki (uM)	Ki (uM)	Ki (uM)
Δ762	0.029 ± 0.004	1.45 ± 0.073	0.074 ± 0.012	0.42 ± 0.14
Δ762^{R1264H}	0.107 ± 0.014	3.81 ± 1.45	0.212 ± 0.017	1.75 ± 0.52
Δ762-1097	0.978 ± 0.084	16.14 ± 15.05	1.716 ± 0.302	12.58 ± 8.47
Δ1118	0.065 ± 0.018	2.19 ± 2.08	0.184 ± 0.064	0.18 ± 0.10
Δ1143	NA	NA	NA	NA

Supplementary Figure 3

Experimental fluorescence anisotropy data used to calculate apparent dissociation constants for RTEL1 deletion constructs binding to RNA and DNA oligonucleotides indicated in Fig. 2. Increasing concentrations of the indicated oligonucleotides were added to reactions containing the indicated RTEL1 proteins and a 24mer FAM-TERRA-MUT RNA at 1 μM. Binding curves used to calculate apparent dissociation constants (Ki) are shown. For RTEL$^{\Delta 1143}$ direct binding to TERRA and TERRA-MUT RNAs is shown. All binding assays were conducted in triplicate and mean and standard deviation are shown.

Supplementary Figure 4

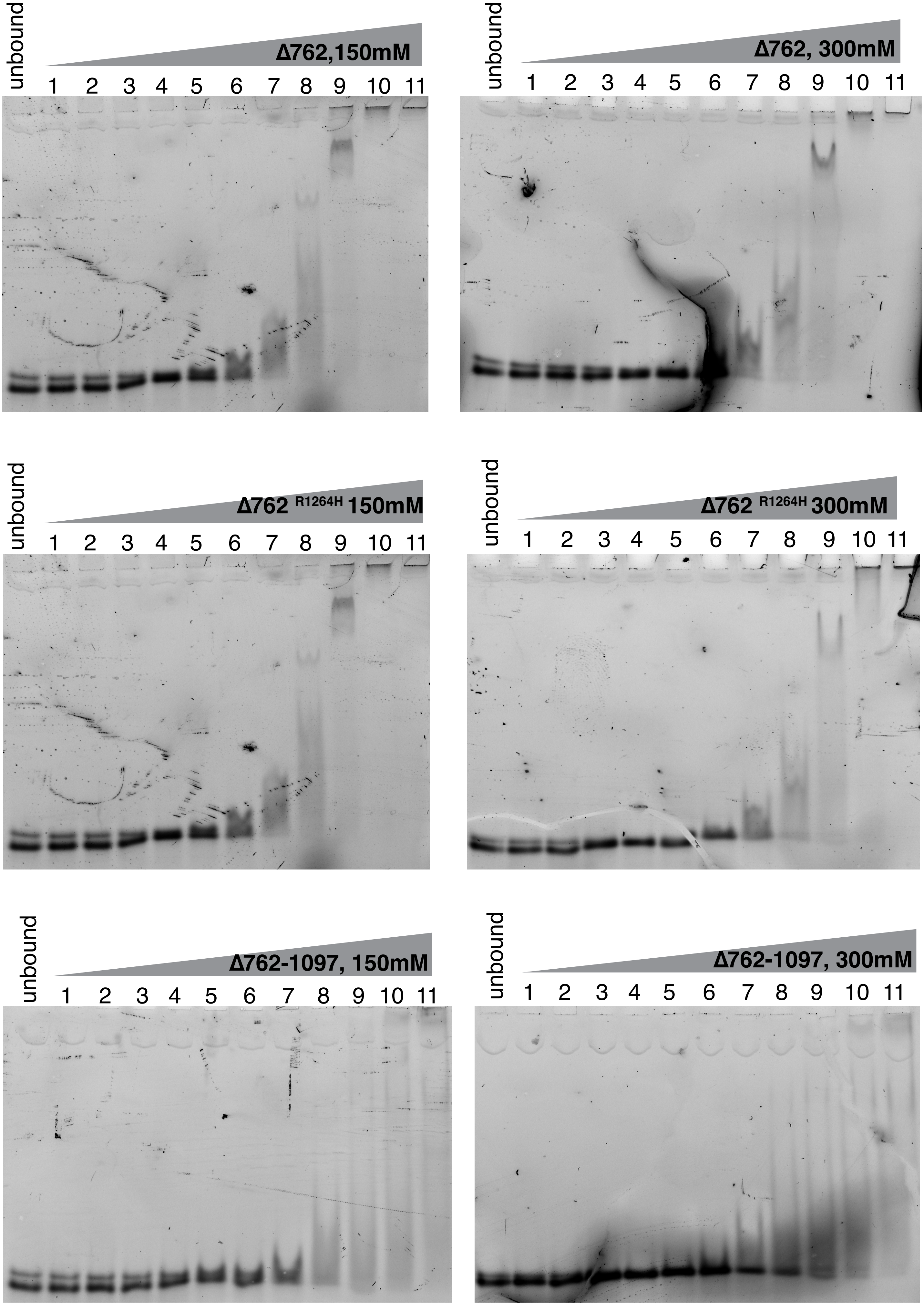

[Protein], nM	
1	0.005
2	0.019
3	0.076
4	0.305
5	1.221
6	4.883
7	19.53
8	78.13
9	312.5
10	1250
11	5000

Supplementary Figure 4

Representative native electrophoretic mobility shift assays (EMSA) with TERRA oligonucleotides at 2.5 nM and increasing amounts of the indicated recombinant proteins. Unbound indicates the free probe without protein added. EMSAs at 150 mM or 300 mM KCl are shown.

Supplementary Figure 5

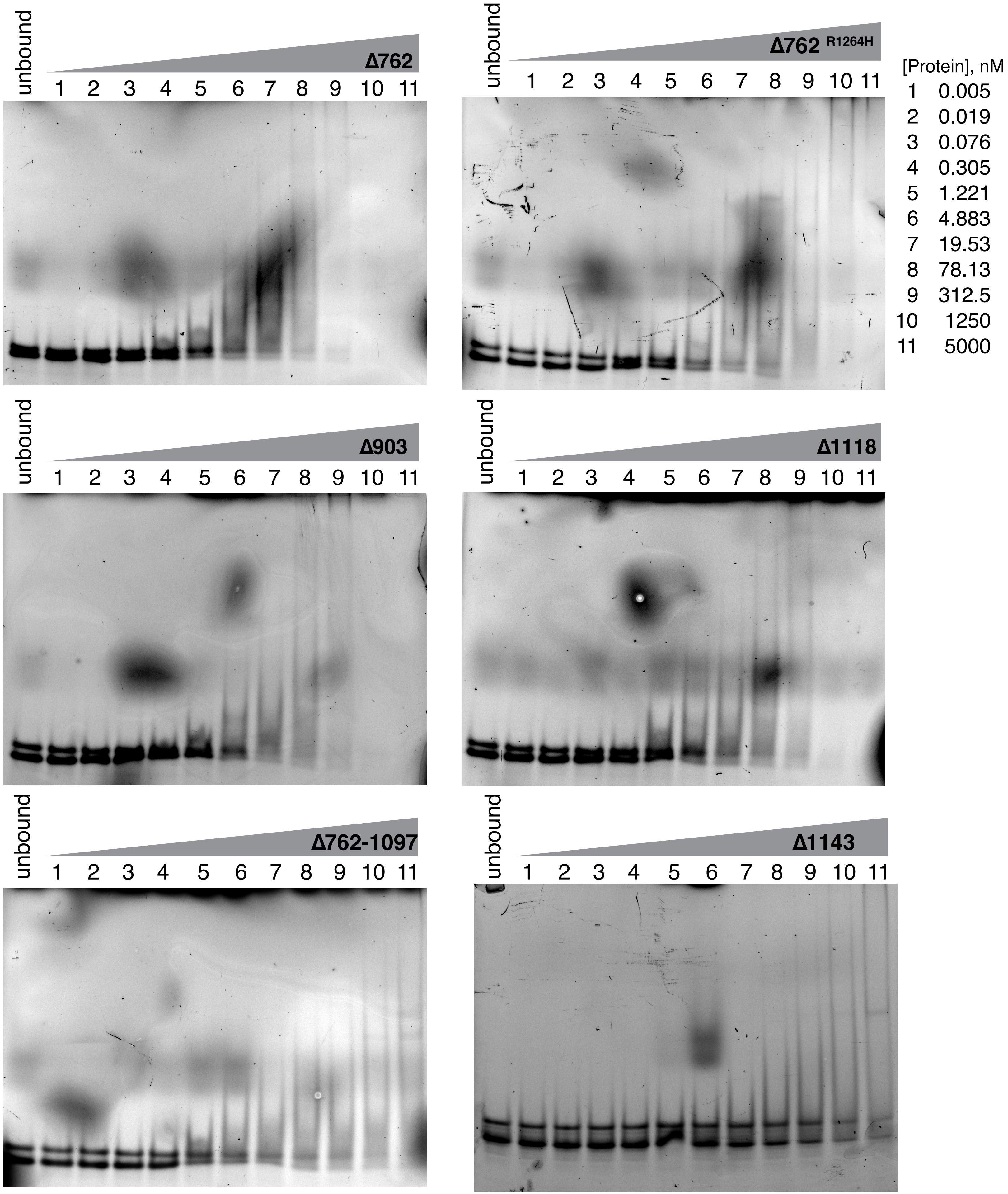

Supplementary Figure 5

Representative native electrophoretic mobility shift assays (EMSA) with TERRA oligonucleotides at 2.5 nM and 150 mM KCl. Increasing amounts of the indicated recombinant RTEL1 truncated proteins were added. Unbound indicates the free probe without protein added. Negligible binding was observed for RTEL1$^{\Delta1143}$ at 5 μM protein concentration.

Supplementary Figure 6

a.

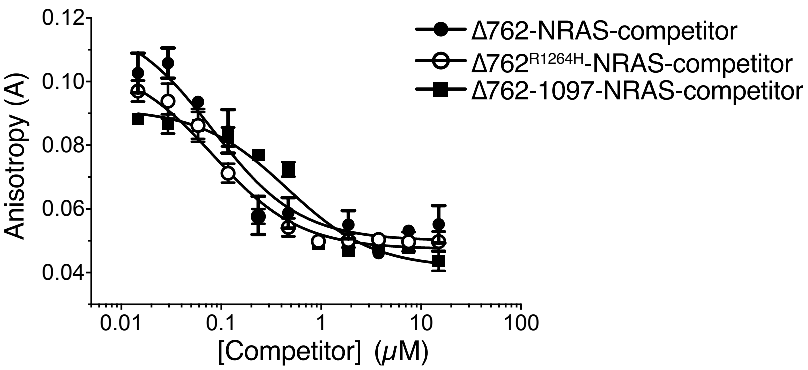

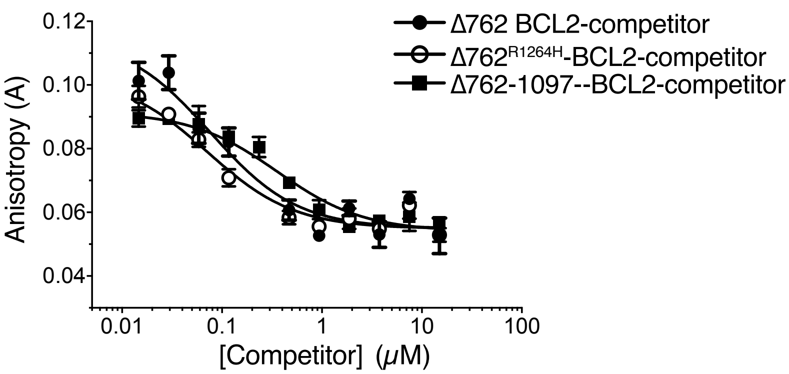

b.

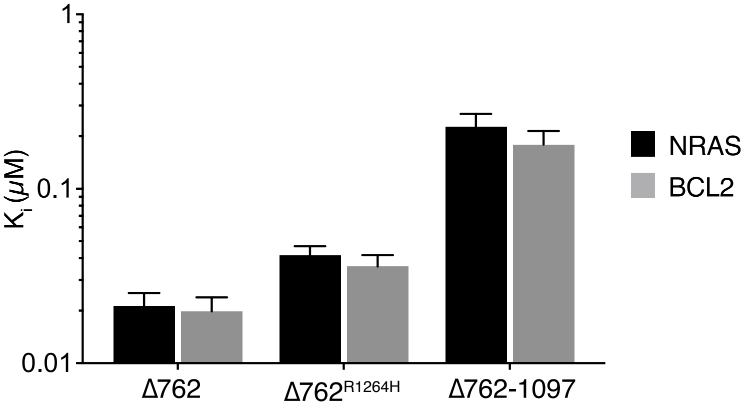

	NRAS	BCL2
	Ki (uM)	Ki (uM)
Δ762	0.021 ± 0.004	0.020 ± 0.004
Δ762^{R1264H}	0.041 ± 0.005	0.036 ± 0.006
Δ762-1097	0.227 ± 0.041	0.179 ± 0.035

c.

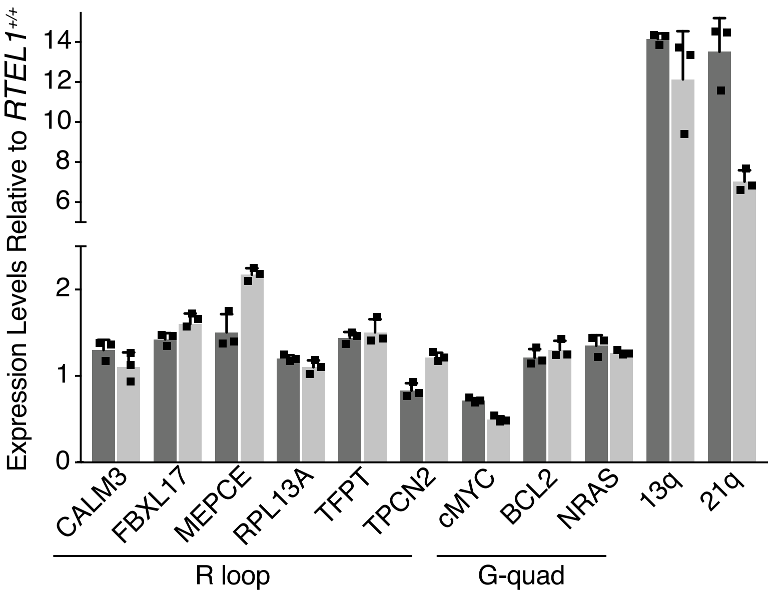

d.

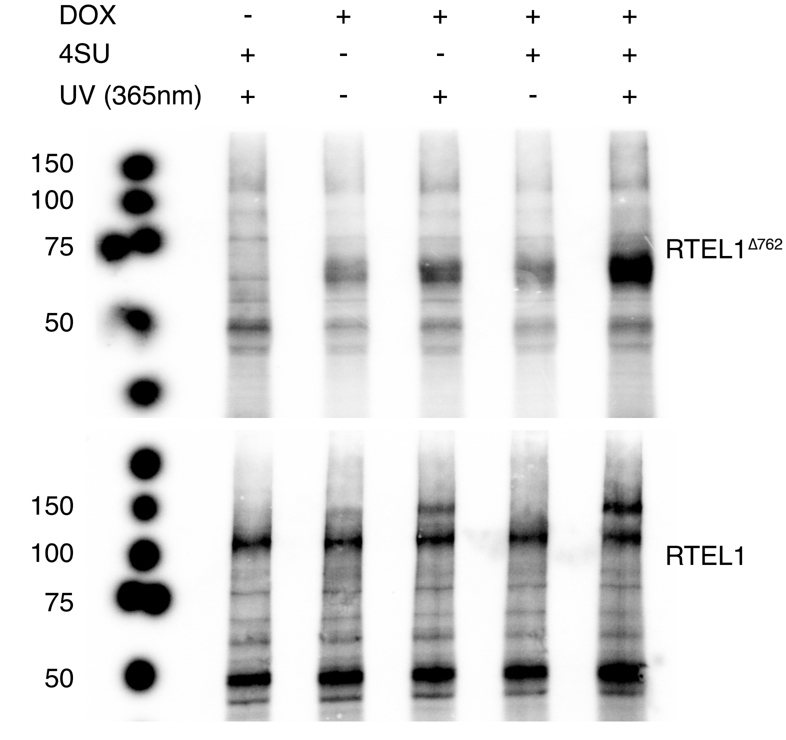

e.

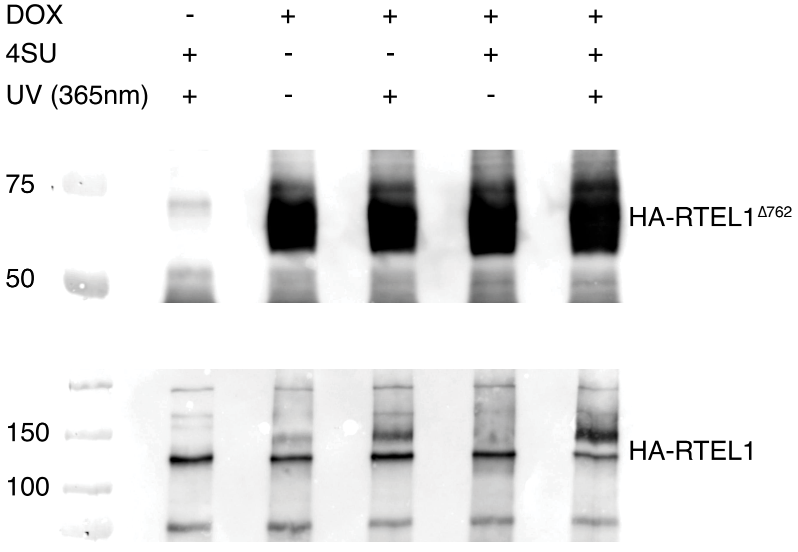

Supplementary Figure 6

RTEL1 binding to NRAS and BCL2 oligonucleotides was monitored by fluorescence anisotropy. (A) Increasing concentrations of the indicated oligonucleotides were added to reactions containing the indicated RTEL1 proteins and a 24mer FAM-TERRA-MUT RNA. Binding curves used to calculate apparent dissociation constants (Ki) are shown. (B) Bar graphs show apparent dissociation constants (Ki) derived by competition experiments in A. All binding assays were conducted in triplicate and mean and standard deviation are shown. (C) Expression levels of non-telomere targets predicted to form R-loops and G-quadruplexes measured by qRT-PCR. No significant differences were observed in RTEL1-KO cells compared to wild type HEK293 cells. Mean and standard deviation are shown for two independent experiments with three technical replicates each. (D) FLAG-tagged RTEL1 and RTEL1$^{\Delta 762}$ proteins were separated by SDS-PAGE after 4SU PAR-CLIP. Autoradiograph of cross-linked, ^{32}P-labelled RNA-FLAG-tagged-RTEL1 immunoprecipitates is shown. (E) Western blot analysis of cross-linked RNA-protein immunoprecipitates shown in D. 4SU PAR-CLIP and Western blots were repeated at least two times yielding similar results. Molecular weight markers, KDa are shown.

Supplementary Figure 7

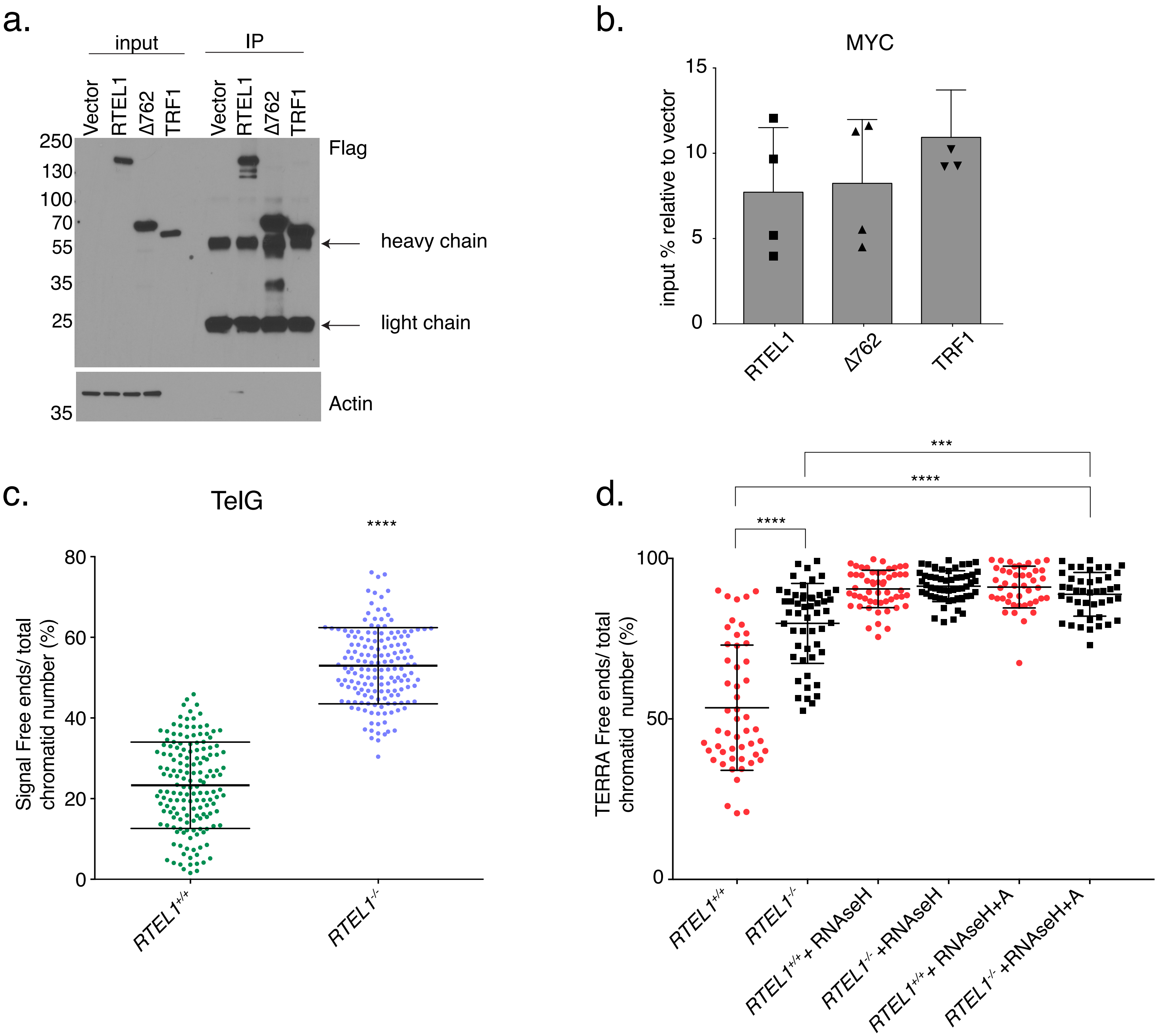

Supplementary Figure 7

(A) Western blot of input and immunoprecipitates of FLAG tagged proteins from RNA-IP samples in Fig. 2E is shown. Molecular weight markers, KDa are shown. (B) Quantitation of 4 independent RNA-IP experiments using a *MYC* probe. The mean and standard deviation of relative RNA-IP normalized to the vector only control is shown. (C) Quantification of telomere loss per chromatid using a TelG PNA probe. Data represents the average of at least 50 metaphases in each of three independent experiments as mean and standard deviation (***$p < 0.001$, two-sided t-test). (D) Quantification of TERRA loss per chromatid following RNAse treatment *in vitro* or treatment with reaction buffer only. Data represents the average of at least 20 metaphases in each of 2 independent experiments as mean and standard deviation ((***$p < 0.001$, ****$p < 0.0001$, two-sided t-test)

Supplementary Figure 8

a.

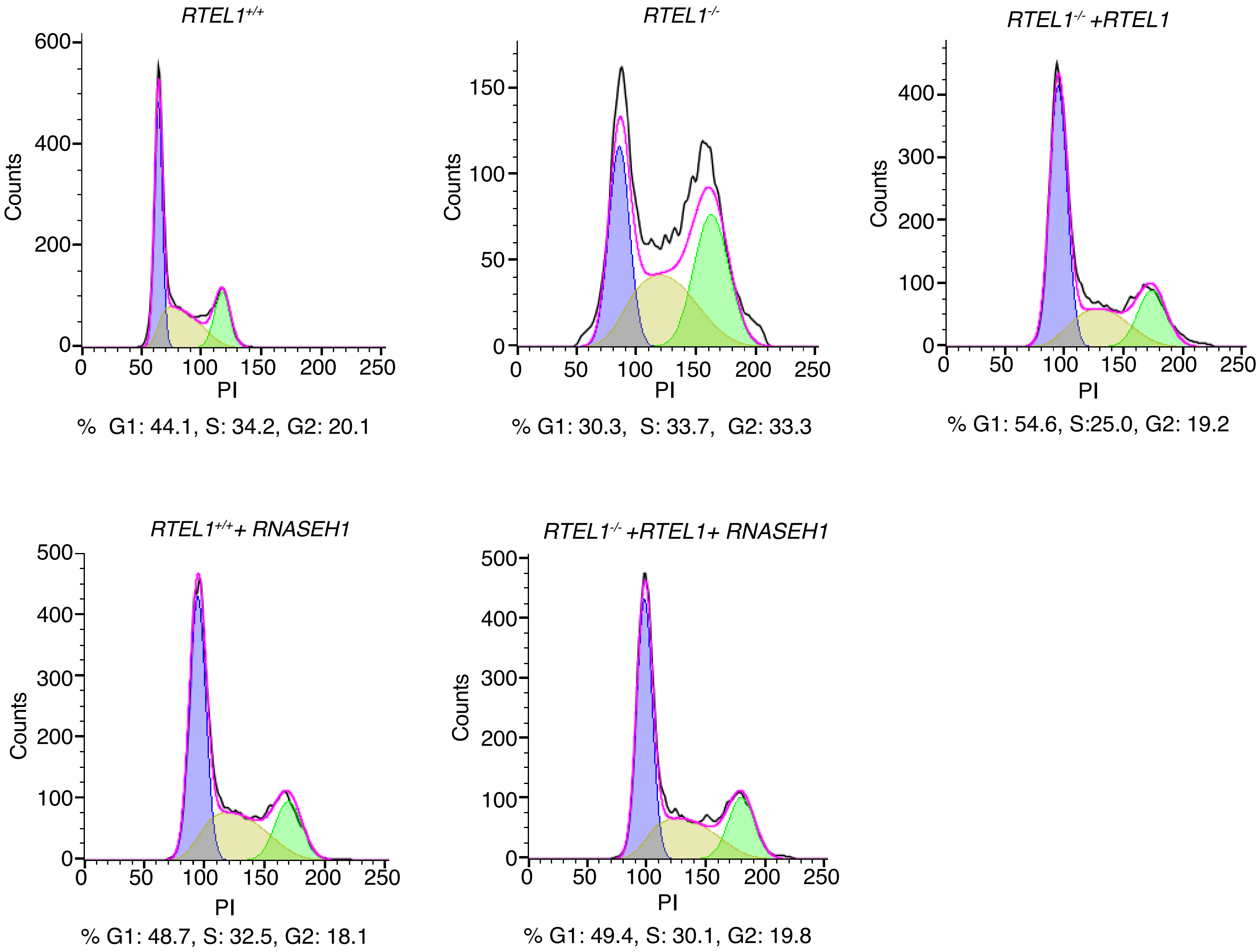

b.

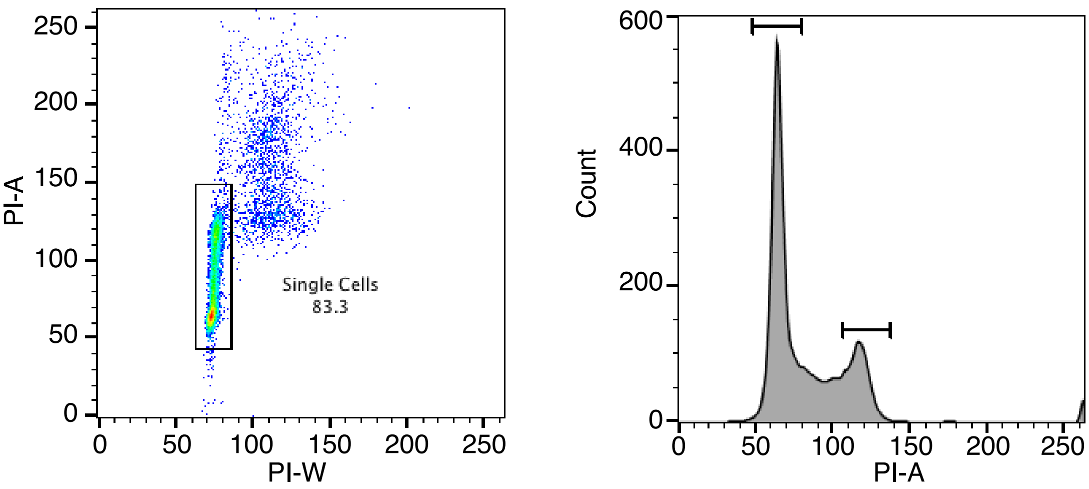

Supplementary Figure 8

Cell cycle analysis was performed by flow cytometry. Cells were fixed, RNase treated, and stained with propidium iodide. (A) Data were analyzed with FlowJo™ software and percentages of cells in different stages of the cell cycle were calculated using the Dean-Jet-Fox model. (B) Representative gating strategy used for flow cytometry analysis is shown. Live cells were gated (SSC/FSC) then singles cells were gated (PI-A/PI-W) and represented as a histogram using PI fluorescence.

Supplementary Figure 9

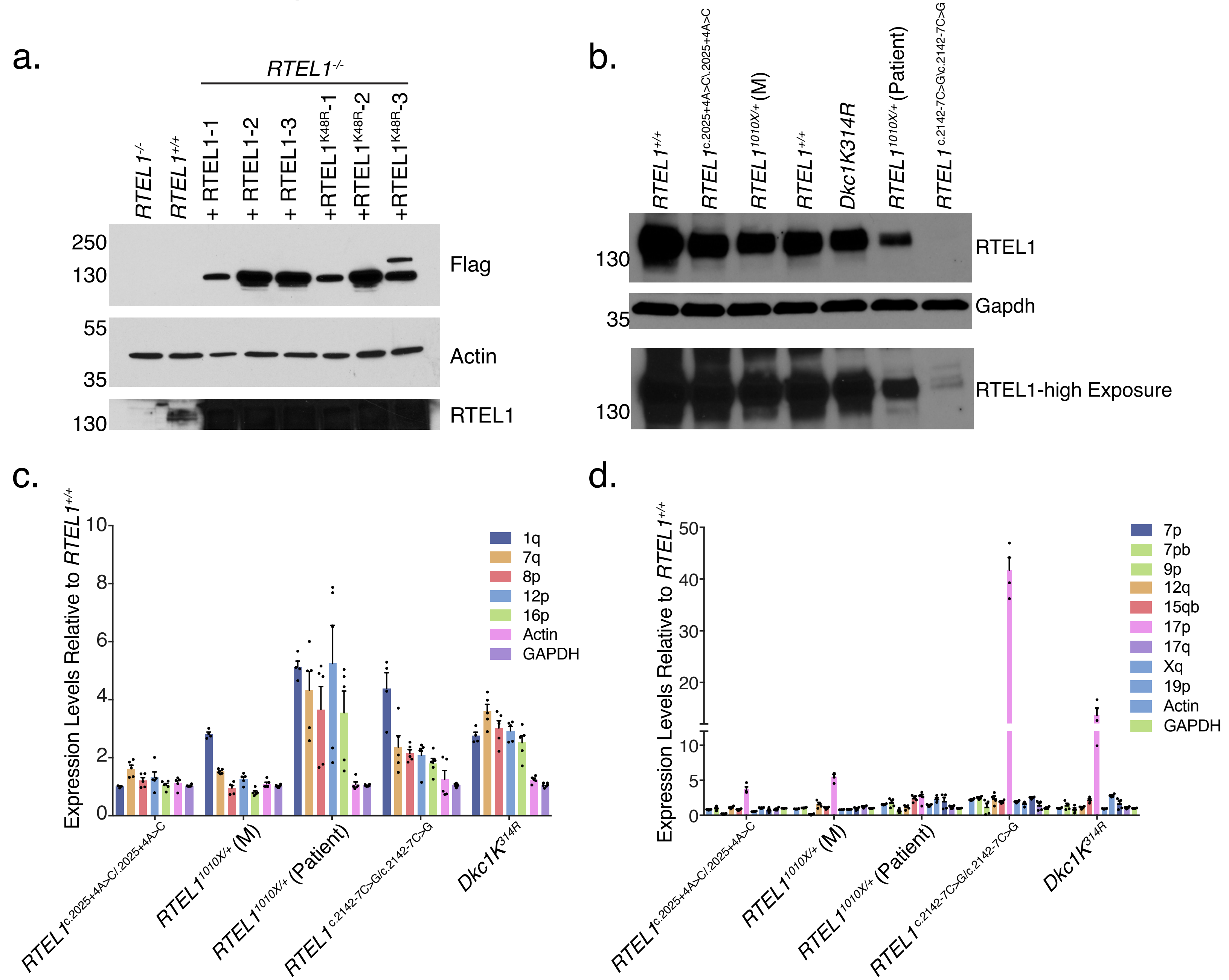

e.

	RTEL1$^{c.2025+4A>C/.2025+4A>C}$	*RTEL1*$^{1010X/+}$ (M)	*Dkc1K*^{314R}	*RTEL1*$^{1010X/+}$ (Patient)	*RTEL1* $^{c.2142-7C>G/c.2142-7C>G}$
10q	***	ns	*	***	*
12p	ns	ns	****	ns	*
13q	ns	**	****	****	***
16p	ns	*	**	*	**
19p	ns	ns	ns	ns	****
21q	ns	***	***	***	****
7q	**	****	****	**	****
8p	ns	ns	**	*	***
9p	****	****	ns	*	ns
10qb	ns	****	****	****	***
12q	ns	ns	ns	ns	**
13qb	ns	***	****	****	***
15qb	*	ns	***	***	***
17p	***	****	***	*	****
17q	****	****	ns	**	****
1q	ns	****	****	****	****
2q	ns	****	****	****	****
7p	***	**	ns	****	****
Xq	*	ns	****	***	**
10qc	***	**	***	****	**
13qc	ns	****	****	****	**
7pb	ns	ns	ns	ns	****
Actin	ns	*	ns	ns	ns
GAPDH	ns	ns	ns	ns	ns

Supplementary Figure 9

(A) Western blot of RTEL1 protein levels in wild type, RTEL1-KO, and three independent clones of *RTEL1-KO HEK293* cells reconstituted with FLAG-RTEL1 and FLAG-RTEL1^{K48R}. Actin was used as loading control, and molecular weight markers, KDa are shown. (B) Endogenous RTEL1 protein levels were measured by western blotting in a panel of patient derived LCL cell lines. Immunoblotting experiments were repeated at least two times producing similar results. TERRA levels are elevated in cells derived from a panel of patient derived LCL lines. (C) Chromosome ends elevated 2.5-5 fold relative to wild type levels are shown. D) Chromosome ends elevated 2.5 fold or under relative to wild type levels are shown and levels for telomeric end 17p which is CTCF regulated is also shown (Beishline, Vladimirova et al. 2017). Levels were measured by qRT-PCR. Values are sample averages of at least 3 experimental repeats with means and standard deviations shown. (E) Calculated p values for TERRA expression measured by qRT-PCR in three independent experiments shown including p values for **Fig. 3E** (*$p < 0.05$, **$p < 0.01$, ***$p < 0.001$, two-sided t-test).

Supplementary Figure 10

a.

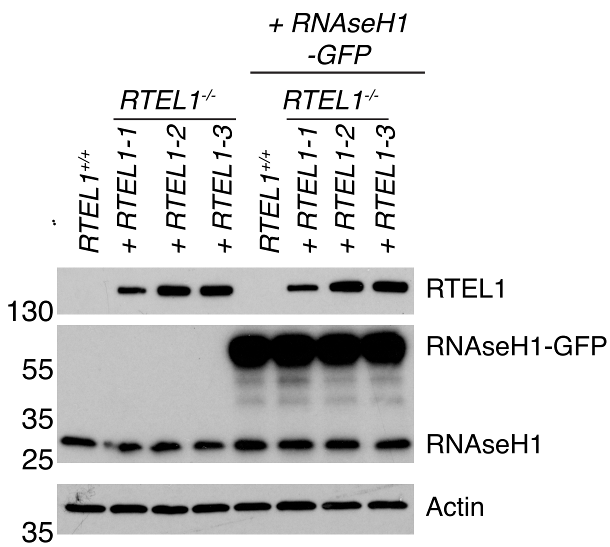

b.

	RTEL1+/++ RNAseH1	RTEL1-/- +RTEL1_1+ RNAseH1	RTEL1-/- +RTEL1_2+ RNAseH1	RTEL1-/- +RTEL1_3+ RNAseH1
10q	***	***	***	**
10qb	***	***	***	**
13q	***	***	***	**
13qb	***	***	***	***
2q	***	***	***	**
21q	***	***	***	***
Xq	n.s	n.s	n.s	n.s
GAPDH	n.s	n.s	n.s	n.s

c.

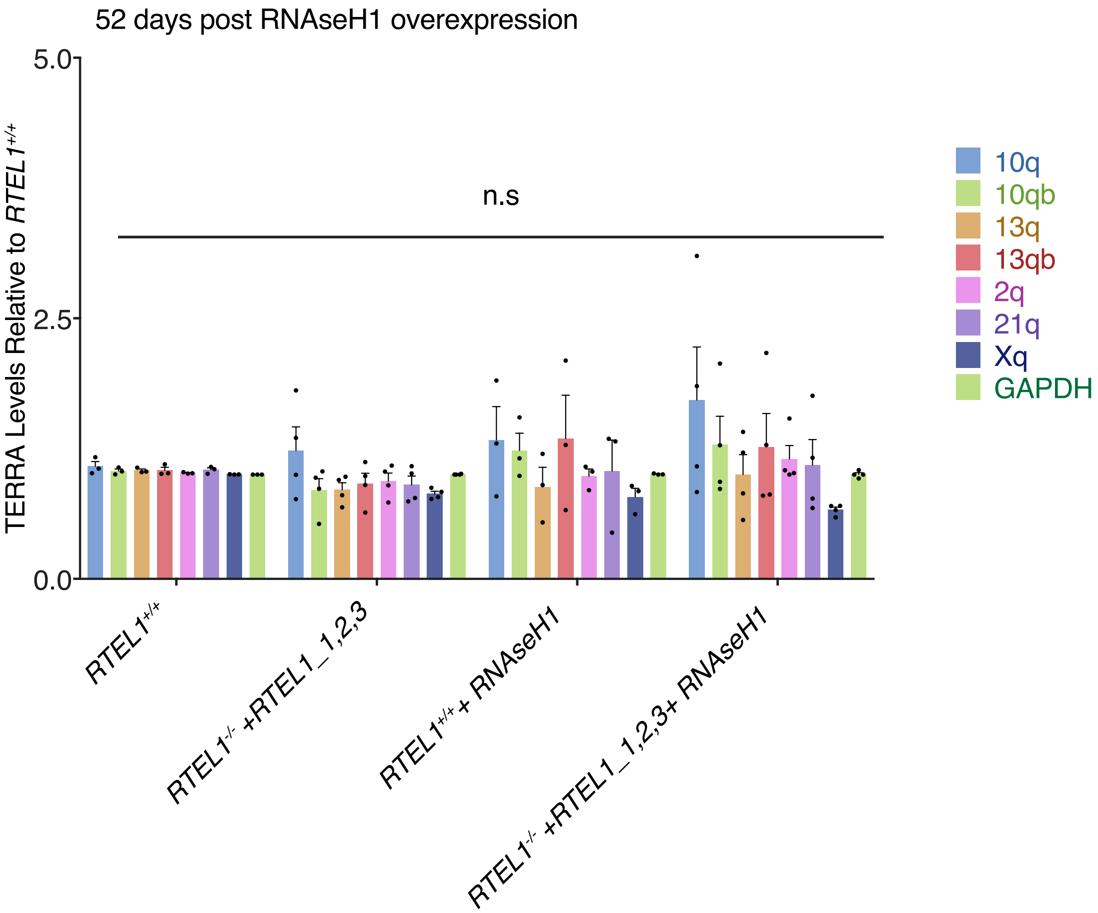

Supplementary Figure 10

(A) Western blotting of wild type HEK293 cells and RTEL1-KO cells complemented with *FLAG-RTEL1* and transfected with *RNAse H1-GFP.* Three independent clones of *RTEL1-KO HEK293* cells reconstituted with FLAG-RTEL1 and with or without *RNAse H1-GFP* are shown. Molecular weight markers, KDa are shown. (B) Calculated p values for TERRA expression measured by qRT-PCR in three independent experiments shown in Fig. 5B (*p < 0.05, **p <0.01, ***p < 0.001, two-sided t-test). (C) TERRA levels 52 days post *RNAse H1* overexpression. TERRA levels of indicated chromosome ends were tested in cells transfected with *RNAse H1-GFP* 52 days post G418 selection. TERRA levels were measured by qRT-PCR. Three independent cell lines complemented with RTEL1 were averaged for analysis. Values are sample averages of at least 3 experimental repeats (*p < 0.05, **p <0.01, ***p < 0.001, two-sided t-test) with mean and standard deviation shown.

Supplementary Table 1

Primer Set	Primer Name	Primer Sequence	Unique	Reference
1	1q_F	GCATTCCTAATGCACACATGAC	yes	M. Feretzaki, J. Lingner / Methods 114 (2017) 39–45
	1q_R	ACCCTAACCCGAACCCTA		
2	2q_F	AAAGCGGGAAACGAAAAGC	no	EMBO J (2012)31:4165-4178 and M. Feretzaki, J. Lingner / Methods 114 (2017) 39–45
	2q_R	GCCTTGCCTTGGGAGAATCT		
3	H2p_F	CGCATCGACG GTGAATAAAA	yes	Sagie, S. et al. T,. Nat. Commun. 8:14015 (2017)
	H2p_R	GCCTAACTCGTG TCTGACTTTGAG		
4	H7q_F	TTCAGACGGG CTTTTGGTTT	yes	Sagie, S. et al. T,. Nat. Commun. 8:14015 (2017)
	H7q_R	ATGGTGAATACA A TCCTTTCTGTTT G		
5	H_7p_F	GGAGGCTGAGGCAGGAGAA	no	EMBO J (2012)31:4165-4178 and M. Feretzaki, J. Lingner / Methods 114 (2017) 39–45
	H_7p_R	CAATCTCGGCTCACCACAATC		
6	H_7p_Fb	GAGAGAGGGTTTCACTCTGTTG	yes	M. Feretzaki, J. Lingner / Methods 114 (2017) 39–45
	H_7p_Rb	GGTGGTTCACGCCTGTAAT		
7	H8p_F	CCGGTTGCAG CCGTTAATA	yes	Sagie, S. et al. T,. Nat. Commun. 8:14015 (2017)
	H8p_R	GGCTTTTGGTTTC CCGTTTT		
8	H9p_F	CGGAAAACGG GAAAGCAAA	no	Sagie, S. et al. T,. Nat. Commun. 8:14015 (2017)
	H9p_R	CGTTCCGACGCT GCAAGT		
9	H10q_F	AACCTGAACC CTAACCCTCC	no	Sagie, S. et al. T,. Nat. Commun. 8:14015 (2017)
	H10q_R	A TTGCAGGGTTC AAGTGCAG		
10	H10q_Fb	AAAGCGGGAAACGAAAAGC	no	EMBO J (2012)31:4165-4178 and M. Feretzaki, J. Lingner / Methods 114 (2017) 39–45
	H10q_Rb	GCCTTGCCTTGGGAGAATCT		
11	H10q_Fc	ATGCACACATGACACCCTAAA	yes	M. Feretzaki, J. Lingner / Methods 114 (2017) 39–45
	H10q_Rc	TACCCGAACCTGAACCCTAA		
12	12q_F	ATTTCCCGTTTTCCACACTGA	no	EMBO J (2012)31:4165-4178 and M. Feretzaki, J. Lingner / Methods 114 (2017) 39–45
	12q_R	CTGTTTGCAGCGCTGAATATTC		
13	H12p_F	AGAACTCTGC TCCGCCTTC	yes	Sagie, S. et al. T,. Nat. Commun. 8:14015 (2017)
	H12p_R	GTTGCGTTCTCTT CAGCACA		
14	H13q_F	CCCGCTTTCCA CACTAAACC	yes	Sagie, S. et al. T,. Nat. Commun. 8:14015 (2017)
	H13q_R	CGCA TCGACAGT GAATAAAATCTT T		
15	H13q_Fb	CCTGCGCACCGAGATTCT	no	EMBO J (2012)31:4165-4178 and M. Feretzaki, J. Lingner / Methods 114 (2017) 39–45
	H13q_Rb	GCACTTGAACCCTGCAATACAG		
16	H13q_Fc	CTGCCTGCCTTTGGGATAA	yes	M. Feretzaki, J. Lingner / Methods 114 (2017) 39–45
	H13q_Rc	AAACCGTTCTAACTGGTCTCTG		
17	H_15q_Fb	TGCAACCGGGAAAGATTTTATT	no	EMBO J (2012)31:4165-4178 and M. Feretzaki, J. Lingner / Methods 114 (2017) 39–45
	H_15q_Rb	GCGTGGCTTTGGGACAACT		
18	H16p_F	AACGGTTCAG TGTGGAAAA T GG	yes	Sagie, S. et al. T,. Nat. Commun. 8:14015 (2017)
	H16p_R	CAACTGGACCCT GCAA TGC		
19	17q_F	GTCCATGCATTCTCCATTGATAAG	no	EMBO J (2012)31:4165-4178 and M. Feretzaki, J. Lingner / Methods 114 (2017) 39–45
	17q_R	AGCTACCTCTCTCAACACCAAGAAG		
20	H_17p_F	GGGACAGAAGTGGATAAGCTGATC	no	EMBO J (2012)31:4165-4178 and M. Feretzaki, J. Lingner / Methods 114 (2017) 39–45
	H_17p_R	GATCCCACTGTTTTTATTACTGTTCCT		
21	H19p_F	GCA TCGACGG TGAATTAAAT CTT	yes	Sagie, S. et al. T,. Nat. Commun. 8:14015 (2017)
	H19p_R	GGCTTTTGGTTTC CCGTTTT		
22	H21q_F	GAATAAAATC TTTCCCGGTTG CT	no	Sagie, S. et al. T,. Nat. Commun. 8:14015 (2017)
	H21q_R	CCGCTTTCCACAC TAAACCATT		
23	H22q_F	CGAAACAGAA CCCGAAGCAG	no	Sagie, S. et al. T,. Nat. Commun. 8:14015 (2017)
	H22q_R	TGCACACA TGAC ACCCAAAA		
24	H_Xyq_F	CCCCTTGCCTTGGGAGAA	no	EMBO J (2012)31:4165-4178 and M. Feretzaki, J. Lingner / Methods 114 (2017) 39–45
	H_Xyq_R	GAAAGCAAAAGCCCCTCTGA		
25	HGAPDH_F	AGCCACATCGCTCAGACAC	na	M. Feretzaki, J. Lingner / Methods 114 (2017) 39–45
	HGAPDH_R	GCCCAATACGACCAAATCC		
26	Hβ-actin F	TCCCTGGAGAAGAGCTACGA	na	Sagie, S. et al. T,. Nat. Commun. 8:14015 (2017)
	Hβ-actin R	AGCACTGTGTTGGCGTACAG		

Supplementary Table 1. qRT-PCR Primers used in this study

Primer sequences used in this study. References for primer validation and if the primer pairs are unique is indicated.
