# Supplementary material for: RTEL1 influences the abundance and localization of TERRA RNA
Source: Nat Commun. 2021 May 21;12:3016. doi: 10.1038/s41467-021-23299-2 (PMC8140157; doi:10.1038/s41467-021-23299-2)
Supplement: Supplementary file 2 — Reporting Summary [file 41467_2021_23299_MOESM2_ESM.pdf]

nature research

Corresponding author(s): John H. J. Petrini

Last updated by author(s): Apr 1, 2021

Reporting Summary

Nature Research wishes to improve the reproducibility of the work that we publish. This form provides structure for consistency and transparency in reporting. For further information on Nature Research policies, see our Editorial Policies and the Editorial Policy Checklist.

Statistics

For all statistical analyses, confirm that the following items are present in the figure legend, table legend, main text, or Methods section.

n/a	Confirmed	
☐	☒	The exact sample size (n) for each experimental group/condition, given as a discrete number and unit of measurement
☐	☒	A statement on whether measurements were taken from distinct samples or whether the same sample was measured repeatedly
☐	☒	The statistical test(s) used AND whether they are one- or two-sided<br>*Only common tests should be described solely by name; describe more complex techniques in the Methods section.*
☒	☐	A description of all covariates tested
☒	☐	A description of any assumptions or corrections, such as tests of normality and adjustment for multiple comparisons
☐	☒	A full description of the statistical parameters including central tendency (e.g. means) or other basic estimates (e.g. regression coefficient) AND variation (e.g. standard deviation) or associated estimates of uncertainty (e.g. confidence intervals)
☐	☒	For null hypothesis testing, the test statistic (e.g. F, t, r) with confidence intervals, effect sizes, degrees of freedom and P value noted<br>*Give P values as exact values whenever suitable.*
☒	☐	For Bayesian analysis, information on the choice of priors and Markov chain Monte Carlo settings
☒	☐	For hierarchical and complex designs, identification of the appropriate level for tests and full reporting of outcomes
☒	☐	Estimates of effect sizes (e.g. Cohen's d, Pearson's r), indicating how they were calculated

Our web collection on statistics for biologists contains articles on many of the points above.

Software and code

Policy information about availability of computer code

Data collection	No software was used for data collection
Data analysis	GraphPadPrism Software version 7, FlowJo version 10.7 used the Dean-Jett-Fox algorithm, Image J version 2.1, Astra 6.1 program (Wyatt Technology), ImageGauge software (GE Healthcare).

For manuscripts utilizing custom algorithms or software that are central to the research but not yet described in published literature, software must be made available to editors and reviewers. We strongly encourage code deposition in a community repository (e.g. GitHub). See the Nature Research guidelines for submitting code & software for further information.

Data

Policy information about availability of data

All manuscripts must include a data availability statement. This statement should provide the following information, where applicable:

- Accession codes, unique identifiers, or web links for publicly available datasets
- A list of figures that have associated raw data
- A description of any restrictions on data availability

Data from this study are available from the corresponding author upon reasonable request. Uncropped images for Western Blots, RNA-IP, and the full protein list for the BioID analysis are provided as source data.

Field-specific reporting

Please select the one below that is the best fit for your research. If you are not sure, read the appropriate sections before making your selection.

- [x] Life sciences
- [ ] Behavioural & social sciences
- [ ] Ecological, evolutionary & environmental sciences

For a reference copy of the document with all sections, see nature.com/documents/nr-reporting-summary-flat.pdf

Life sciences study design

All studies must disclose on these points even when the disclosure is negative.

Sample size	Sample sizes were chosen according to standards in the field (Deng, Norseen et al. 2009, Lopez de Silanes, Stagno d'Alcontres et al. 2010, Porro, Feuerhahn et al. 2014, Beishline, Vladimirova et al. 2017, Feretzaki and Lingner 2017, Sagie, Toubiana et al. 2017, Feretzaki, Renck Nunes et al. 2019 and others) . Proper negative and positive controls were used when possible. The number of independent experiments is indicated in the figure legends.
Data exclusions	No data were excluded
Replication	All experiments were performed at least in triplicate an individual points for each repeat is shown for all bar graphs. When indicated experiments were repeated at least 2 times. For all experiments attempts at replication were successful.
Randomization	samples were chosen randomly
Blinding	For all FISH experiments image acquisition and analysis was blinded. For the rest of the experiments blinding was not applicable due to the nature of the experiment (data analysis was done using software such as Prism 7 and not counted by the researcher)

Reporting for specific materials, systems and methods

We require information from authors about some types of materials, experimental systems and methods used in many studies. Here, indicate whether each material, system or method listed is relevant to your study. If you are not sure if a list item applies to your research, read the appropriate section before selecting a response.

Materials & experimental systems

n/a	Involved in the study	
[ ]	[x]	Antibodies
[ ]	[x]	Eukaryotic cell lines
[x]	[ ]	Palaeontology and archaeology
[x]	[ ]	Animals and other organisms
[x]	[ ]	Human research participants
[x]	[ ]	Clinical data
[x]	[ ]	Dual use research of concern

Methods

n/a	Involved in the study	
[x]	[ ]	ChIP-seq
[ ]	[x]	Flow cytometry
[x]	[ ]	MRI-based neuroimaging

Antibodies

Antibodies used	The antibodies used in this study were GFP JL-8 (Clonetech, 632381, 1:4000), ANTI-FLAG® M2 (Sigma, F1804, 1:1000), HA (Biolegend #901515, 1:1000), myc 9E10 (BioXcell, BE0238, 1:4000), RTEL1 (custom made 1:2000-5000), Actin-HRP (ABCAM, ab49900 1:50000), GAPDH (Santacruz, sc_32233, 1:1000) and RNAse H1 (Proteintech 15606-1-AP, 1:1000).
Validation	The specificity of the custom made human RTEL1 antibody was tested upon disappearance of the corresponding RTEL1 band upon CRISPR knockout and in over-expression conditions (Figures 3B, S9A,S9B). Commercially available antibodies are commonly used and below are three examples for each antibody. Antibodies are also validated for western blotting by each of the manufacturers, and were also validated in this manuscript by western blot in endogenous and overexpression conditions.<br><br>GFP JL-8 (Clonetech, 632381, 1:4000): Nat Commun. 2017;8:14907, Proc Natl Acad Sci U S A. 2017;114:E7803-E7811, Nature communications. 2016;7:10811<br><br>ANTI-FLAG® M2 (Sigma, F1804, 1:1000): Nature communications, 6, 6253 (2015-2-24), Nature communications, 8, 16017 (2017-7-7), Nucleic Acids Research 45(7), 4189-4201, (2017)<br><br>HA (Biolegend #901515, 1:1000): J Neurosci. 23:5561, J Cell Biol. 153:649,<br>J Biol Chem. 275:37712

myc 9E10 (BioXcell, BE0238, 1:4000, J Immunol 194(3): 1080-1089, Immunity 42(1): 68-79, Mol Cell 51(1): 46-56

Actin-HRP (ABCAM, ab49900 1:50000): Nat Commun 11:1775 (2020), Front Oncol 10:316 (2020), J Neuroinflammation 17:22 (2020).

GAPDH (Santacruz, sc_32233, 1:1000): Front Immunol. 12: 628117, J Biol Chem. 100382, RNA Biol. 1-16

RNAse H1 (Proteintech 15606-1-AP, 1:1000): Nucleic Acids Res 2019 4;47(10):5086-5099, Sci Rep 2019 13;9(1):19110, PLoS Genet . 2015 19;11(11):e100567

Eukaryotic cell lines

Policy information about cell lines

Cell line source(s)	Flp-In T-REx HEK293 cells Thermo Fisher Scientific R78007 and HEK293T Dharmacon #HCL4517
Authentication	Parental Flp-In-293 cell line was obtained commercially and validated in (Garzia, Jafarnejad et al. 2017, Garzia, Meyer et al. 2017). All cell lines were authenticated by Short Tandem Repeats (Garzia, Jafarnejad et al. 2017, Garzia, Meyer et al. 2017). Overexpression cell lines were authenticated by western blotting. CRISPR'Cas9 KO cells were authenticated by western blotting and sequencing.
Mycoplasma contamination	Cells were monitored for mycoplasma on a monthly basis and tested negative.
Commonly misidentified lines (See ICLAC register)	no commonly misidentified cell lines were used in the study.

Flow Cytometry

Plots

Confirm that:

- [x] The axis labels state the marker and fluorochrome used (e.g. CD4-FITC).
- [x] The axis scales are clearly visible. Include numbers along axes only for bottom left plot of group (a 'group' is an analysis of identical markers).
- [x] All plots are contour plots with outliers or pseudocolor plots.
- [x] A numerical value for number of cells or percentage (with statistics) is provided.

Methodology

Sample preparation	Cells were harvested by trysinization and fixed with cold 70% ethanol overnight. Fixed cells were washed with 1X PBS and stained with 2mg/ml propidium iodide and 10mg/ml RNAse A in PBS.
Instrument	Fortessa 3
Software	FlowJo™
Cell population abundance	The Dean-Jett-Fox algorithm FlowJo™ was used to calculate the percentage of cells in each phase of the cell cycle
Gating strategy	Live cells were gated, doublets were excluded and PI fluorescence was analyzed.

- [x] Tick this box to confirm that a figure exemplifying the gating strategy is provided in the Supplementary Information.
