# Supplementary material for: RTEL1 influences the abundance and localization of TERRA RNA
Source: Nat Commun. 2021 May 21;12:3016. doi: 10.1038/s41467-021-23299-2 (PMC8140157; doi:10.1038/s41467-021-23299-2)

Source Data

Box shows lanes used

Figure 1B left uncropped blots

Input IP

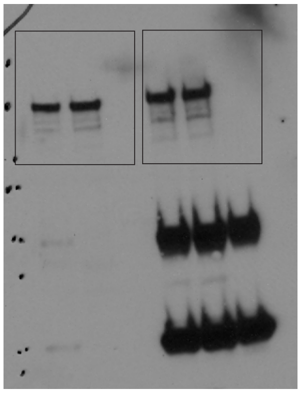

MYC

Input

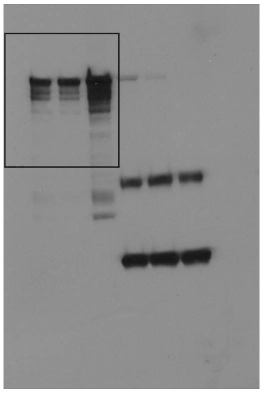

Flag

IP

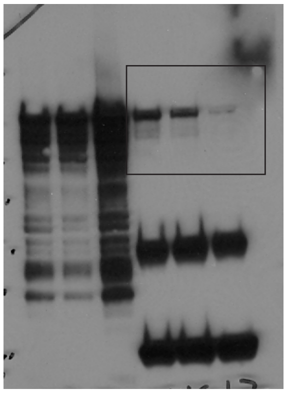

Flag

Figure 1B center uncropped blots

Input IP

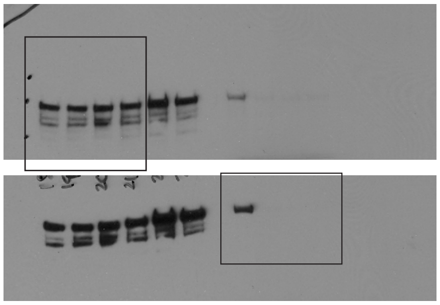

MYC

Input IP

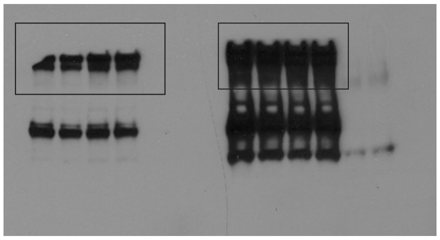

GFP

Figure 1B right uncropped blots

Input IP

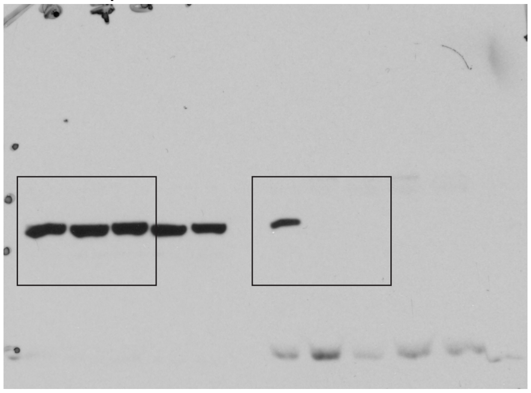

Flag

Input IP

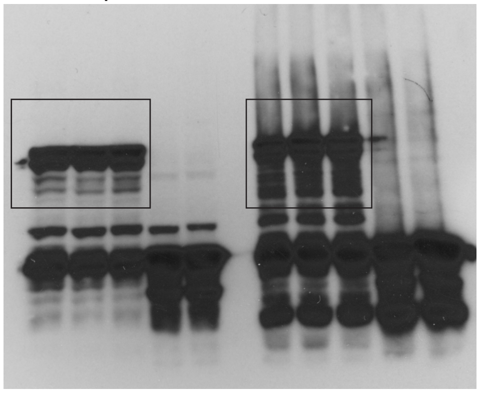

GFP

Box shows lanes used

Figure 2E uncropped image

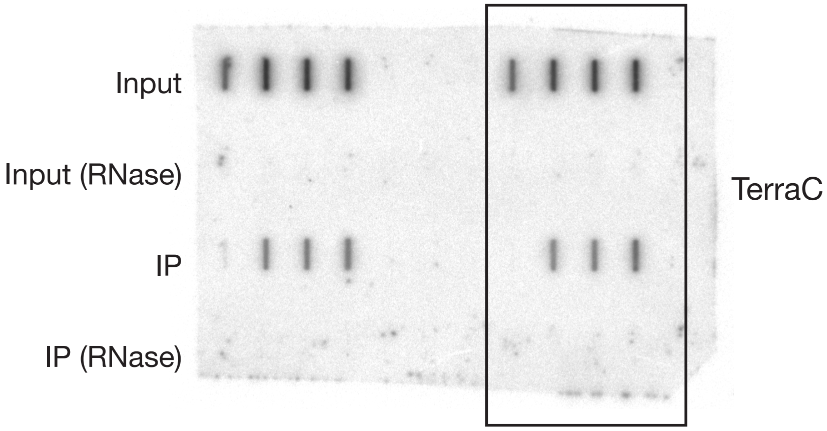

Figure 3B uncropped blots

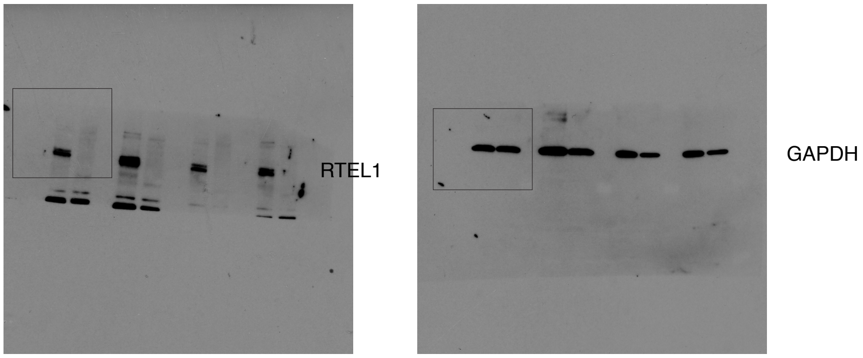

Figure S6 D uncropped radiographs

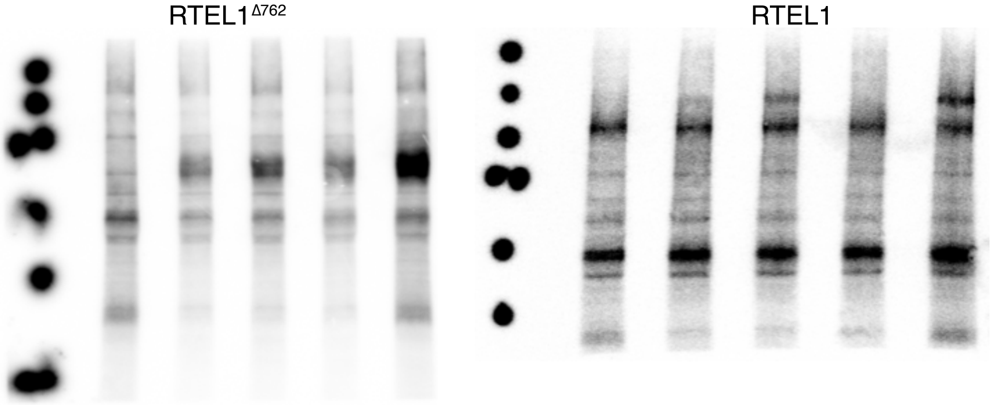

Figure S6 D uncropped cut membranes and blot

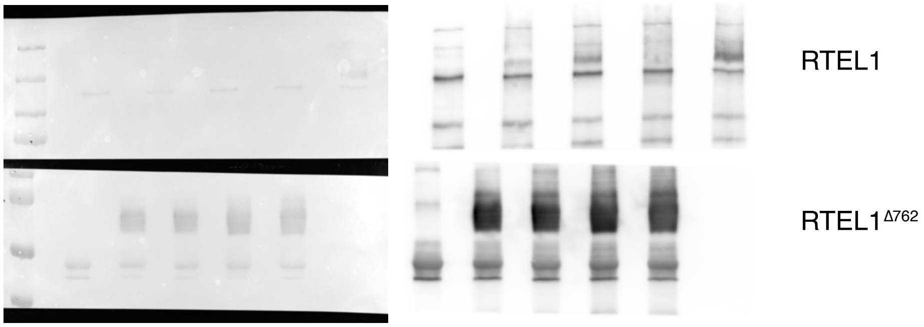

Box shows lanes used

Figure S7 A uncropped blots

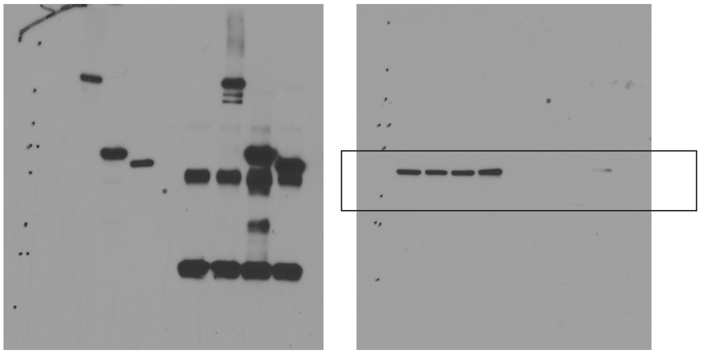

Figure S9 A uncropped blots

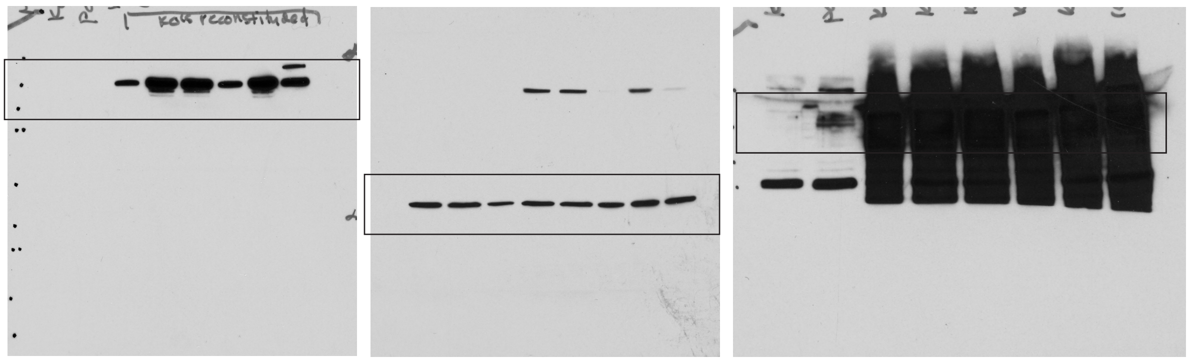

Flag RTEL1 | ACTIN | RTEL1

Figure S9 B uncropped blots

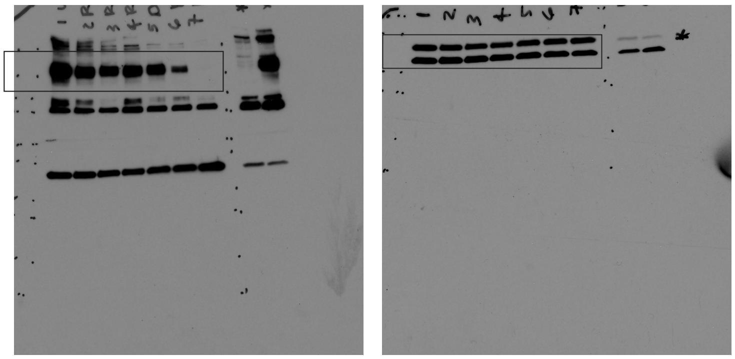

RTEL1

GAPDH: bottom band used in figure
ACTIN: top band not used in figure

Figure S10 A uncropped blots

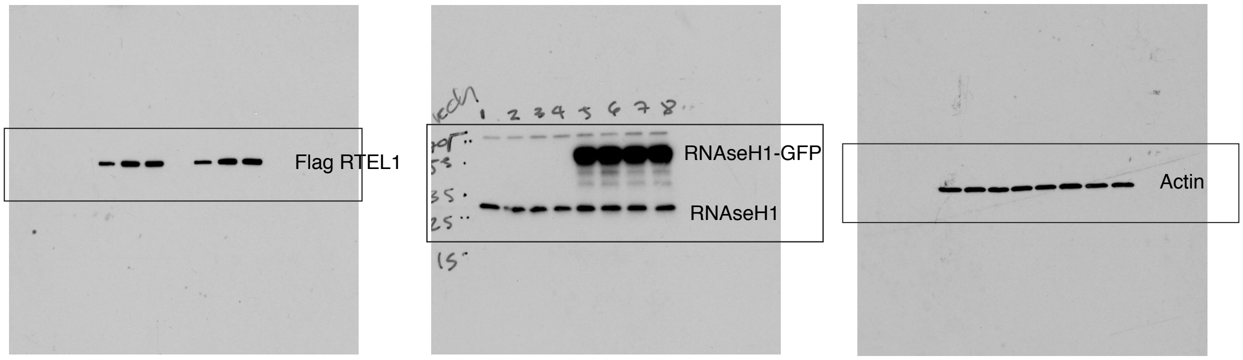

Supplement: Supplementary file 3 — Source Data [file 41467_2021_23299_MOESM3_ESM.zip › 260726_3_supp_5508690_qrvncs.pdf]
